# Seed and foliar application of nano-selenium improves sesame triacylglycerols and oil yield via photosynthetic pigment and enzymatic and chemical antioxidant enhancement revealed by spectrophotometric, UHPLC-analysis and chemometric modeling

**DOI:** 10.3389/fpls.2024.1431877

**Published:** 2024-11-05

**Authors:** Ilyas Ahmad, Chi Chen, Zohaib Younas, Tayyaba Yousaf, Zia-ur-Rehman Mashwani

**Affiliations:** ^1^ Department of Botany, Arid Agriculture University, Rawalpindi, Punjab, Pakistan; ^2^ Department of Food Science and Nutrition, College of Food, Agriculture and Natural Resources, University of Minnesota, Minneapolis, MN, United States; ^3^ Pakistan Academy of Sciences, Islamabad, Pakistan

**Keywords:** TS-5, V2: TH-6, V3: Til-18, V4: Niab Millennium, V5: Niab Pearl, antioxidant, UHPLC-MS, chemometric

## Abstract

The current study aimed to investigate the effects of plant-mediated selenium nanoparticles (SeNPs) on plant growth, photosynthetic pigments, antioxidant activity, and the triacylglycerol profile of sesame (*Sesamum indicum* L.). The green synthesis of SeNPs was achieved using garlic extract, resulting in spherical nanoparticles with an average size range of 70–75 nm. Three SeNP treatments (T3, 30 ppm; T4, 40 ppm; and T5, 50 ppm) were applied through seed and foliar spray on six sesame varieties (V1, TS-5; V2, TH-6; V3, Til-18; V4, Niab Millennium; V5, Niab Pearl; and V6, NS-16). All enzymatic antioxidant parameters showed an increase in the treated groups, such as SOD (74.4% in V1 at T4), POD (43% in V5 at T5), APX (62% in V1 at T3), and GPX (31.56% in V3 at T4). CAT showed the highest percentage improvement in T5 for V1, V2, V4, and V5, while V3 and V4 exhibited the highest values at T4. Likewise, seed antioxidant parameters also showed increase in antioxidant activity, highest total phenolic content (6.06 mg GAE/g) was found at T5 treatment with percent increase of 27.41%, but the highest percent increase was found to be at T4 treatments in V1 with increase of 46.83%. Percent oil yield was also noted to be higher as highest percent (60%) oil yield was obtained at T4 treatment in V3. Ultra High Performance Mass-Spectrometry (UHPLC-MS) analysis and chemometric modeling suggested a total of 10 triacylglycerol (TG) biomarkers separating untreated groups, with higher relative abundance values at T4 and T5 treatments compared to control. PCA and correlation analysis showed clustering of untreated groups from T4 and T5, which suggests that these two treatments result in higher accumulation of oil. A generalized linear model with ANOVA showed a highly significant impact of treatments on all the growth and oil parameters, with significance involvement of varieties. The interaction between variety and treatment showed no significant effect on the growth and oil biomarkers of sesame. However, it can be concluded that the T4 and T5 treatments (40 ppm and 50 ppm) of SeNPs, applied through seed and foliar methods, have a strong influence on the overall growth and oil yield of sesame. This warrants further transcriptomic and molecular analysis to gain deeper insight into the mechanisms of action of SeNPs.

## 1 Introduction

Sesame (*Sesamum indicum* L.), the first oil seed crop known to be consumed by humans and domesticated approximately 3,000 years ago, is often referred to as the “Queen of Oilseeds” due to its high oil content. Despite this, sesame is considered an “orphan crop” because it has received significantly less research attention compared to other oil seeds and staple crops. However, the demand for sesame seed oil (SSO) has been increasing due to its high oil (44-55%) and protein (18%–25%) content, antioxidant properties, and beneficial fatty acid composition (Myint et al., 2020). Sesame seeds are not only used in a wide range of edible products in both raw and roasted forms but also used in cosmetics, soaps, lubricants, animal feed, and pharmaceutical uses (Bedigian, 2010). It contains high amount of tryptophan and methionine, fibers, along with diversity of secondary metabolite, i.e., flavonoids, phenols, vitamins (B and E), saponins, and lignans, along with sufficient amounts of nutrients like iron, calcium, and phosphorus. As it contains 83%–85% unsaturated fatty acids like oleic and linoleic acid along with natural antioxidant like sesamin and sesamol, which not only prevents rancidity but also make it a balanced nutritional candidate (Wei et al., 2015), resulting in increasing demand of sesame seeds. Due to changing consumer lifestyle and health awareness, sesame seed consumption is expected to reach $7,245 million by the end of 2024, compared to $6,559 in 2018 (Abdellatef et al., 2008). Tanzania is the leading sesame exporter followed by China, Myanmar, and India; Pakistan became the fifth largest exporter in the world in 2024 due to changing patterns of cash crop by the farmers, with exports of $400 million this year.

Pakistan is suffering from high demand and supply gap of edible oil since 1970s, importing approximately 88.6% to bridge the gap, with only 11.4% of local production (Ahmad et al., 2022). Population explosion and high edible oil consumption resulted in increased per capita utilization from 5.3 kg in 1973 to 20 kg in 2018, which is likely to reach 22 kg in near 2028, widening the demand and supply gap (Rana et al., 2022). The gap in demand and supply made the country from self-sufficient to major edible oil importer in the previous few decades, indicating laps in the policy failures.

Conventional agricultural practices, which relies on bulky doses of fertilizers, pesticides, and insecticides, have negatively impacted the environment resulting in the spread of a wide array of chemical species in soil and atmosphere. Furthermore, sustainable agriculture also requires that it should be a low input system, with reduced production costs and higher net returns (Singh et al., 2021). Similarly, there is a dire need to enhance the agricultural production in an ecofriendly manner, so that agrochemical usage can be reduced to protect the ever-increasing environmental pollution. Here comes the twenty-first century science “Nanotechnology,” which operates in matters at nanoscale. These nanosized particles having higher surface area to volume ratio are more potent and efficient as compared to bulk-sized particles, which make them a good candidate compared to traditional practices used to improve agricultural production. A variety of nanotechnology-based products, such as nanopesticides, nanofertilizers, nanoinsecticides, nanoemulsions, and nanoparticles, has revolutionized the agriculture sector with improving the production with reduced use of toxic chemical species (Mali et al., 2020). Nanoparticles are being synthesized by chemical, physical, and green methods; while the first two methods require special equipment and expensive chemical, green methods only requires small quantity of plant extract and precursor salt, resulting in an environment-friendly plant-based nanoparticles (El-Saadony et al., 2021).

Selenium (Se), once considered a toxic element for plants and animals, is now recognized as an essential element performing crucial functions in both plants and humans. In plants, selenium has been shown to promote growth and mitigate the negative effects of both biotic and abiotic stresses (Mangiapane et al., 2014). Furthermore, Se carries the role as pro-oxidant or antioxidant depending upon the dose and method of application (Jampílek and Kráľová, 2017). Selenium nanoparticles (SeNPs), in contrast to its bulky precursor salt, has high surface area, improved solubility, potency, higher activity, and surface chemistry, which got them more attention to be used as a crop ameliorator. These properties determine their bioavailability, stability, controlled release, and multifunctionality as compared to selenium salts. Furthermore, it has also been proved that biologically synthesized SeNPs are environment friendly, cost effective, and non-toxic; this method can easily be transformed to large-scale production (Hernández-Díaz et al., 2021).

Pakistan’s national food policy is mainly concerned with staple crops like wheat, in which farmers are incentivized and get price support from the government. Most of the oil seed crops being Rabi or winter crops, farmers opt for wheat over oil seeds because of certain demand and prices. Therefore, most of the research work is also attributed to staple crops, neglecting oil seeds crops although with comparatively more profitable. Hence, a lot of data are available regarding the effect of nanoparticles on staple crops, but oil seed crops, especially sesame, has a negligible research regarding oil improvement with nanoparticles. Furthermore, most of the studies are limited to one or two germplasms of sesame, but there are mainly six varieties, which are sown in different areas of Pakistan, depending upon climate and geographical conditions.

Therefore, this study was designed to assess the profound influence of plant-mediated selenium nanoparticles on growth and oil yield of sesame. We scrutinized six prominent varieties of sesame, chosen for their cultivation across diverse regions of Pakistan. These varieties exhibit discernible disparities in high-temperature tolerance, branching patterns, stress resilience, and yield potential. Given the potential variance in response to varying concentrations of selenium nanoparticles among these germplasms, our investigation aimed to unravel the interplay between sesame varieties and SeNPs concentrations, offering invaluable insights into their interactive dynamics. The interactive effect of SeNP treatments and varieties was further evaluated to check which one factor contributes more to varying response to SeNPs.

## 2 Materials and methods

### 2.1 Plant extract preparation and synthesis of nanoparticles

Approximately 25 g of garlic (*Allium sativum*) powder was dissolved in 250 mL of distilled water, heated at 120°CC, for 30 min, and the extract was filtered thrice to get the clear plant extract and stored at 4°CC till further use. Approximately 0.43 g of precursor salt (sodium selenite) was dissolved in 500 mL of distilled water, heated and stirred for 15 min, followed by gradual addition of garlic extract (40-60 mL). Solution was constantly heated (155°CC) and stirred with a magnetic stirrer for 6–7 h, and placed in darkness for 4 days. As the reduction process of SeNPs is slow, it requires darkness for approximately 3–4 days for complete reduction, which is indicated by brick red color. The resulting solution was centrifuged at 14,000 rpm, the supernatant was discarded, the pellets were washed with methanol, and the resulting SeNPs were placed in an incubator at 60°CC for 2 days. After 2 days, SeNPs were scratched from Petri plates and sonicated for 40 min with a sonicator, and the resulting SeNPs were used for crop application and characterization (Ahmad et al., 2023).

### 2.2 Characterization of plant-mediated SeNPs

The characterization of SeNPs was carried out with different analytical techniques. Color change from white to yellow and ultimate brick red was the first indictor considered for confirmation of SeNPs. Furthermore, UV spectroscopy was employed at 200–800 nm using a UV 752 spectrophotometer; absorption data were acquired, and line plot was drawn to check the highest absorption peaks. Morphological analysis of SeNPs was done with scanning electron microscopy (JSM5910, JEOL, Japan) with energy range of 30 kV with ×10k magnification. Drop coating method was used to make a sample on carbon-coated grid. Sample drying was achieved by putting it under the mercury lamp for 10 min, and a bloating paper was used to remove extra solution. Different magnification powers were used to check the morphology of selenium nanoparticles (Javed and Mashwani, 2020). Fourier transform infrared spectroscopy (FTIR) is an important characterization technique, which is used to check the functional groups present on the surface of nanoparticles. As in this study, garlic extract was used; FTIR was carried out to check the functional groups responsible for the reduction and stabilization of SeNPs. The dried powder of SeNPs were compressed alongside potassium bromide, and the FTIR spectrum obtained using a Perkin-Elmer FTIR-Spectrum instrument (USA) transmittance was acquired within the wavenumber range of 500–4000 cm^−1^. X-ray diffraction analysis of SeNPs was carried out at 40 kV and 30 mA within 2θ area between 20°C and 80°C with radiation intensity Cu-Ka (λ = 0.15406 nm) with a Siefert X-ray diffractometer. In order to check and confirm the elemental analysis of SeNPs, energy dispersive X-ray (EDX) was conducted using an EDX device. The procedures previously outlined were followed for the preparation and analysis of the samples (Javed et al., 2020).

### 2.3 Seed collection, SeNP treatments, and experimental layout

**Figure 1:**
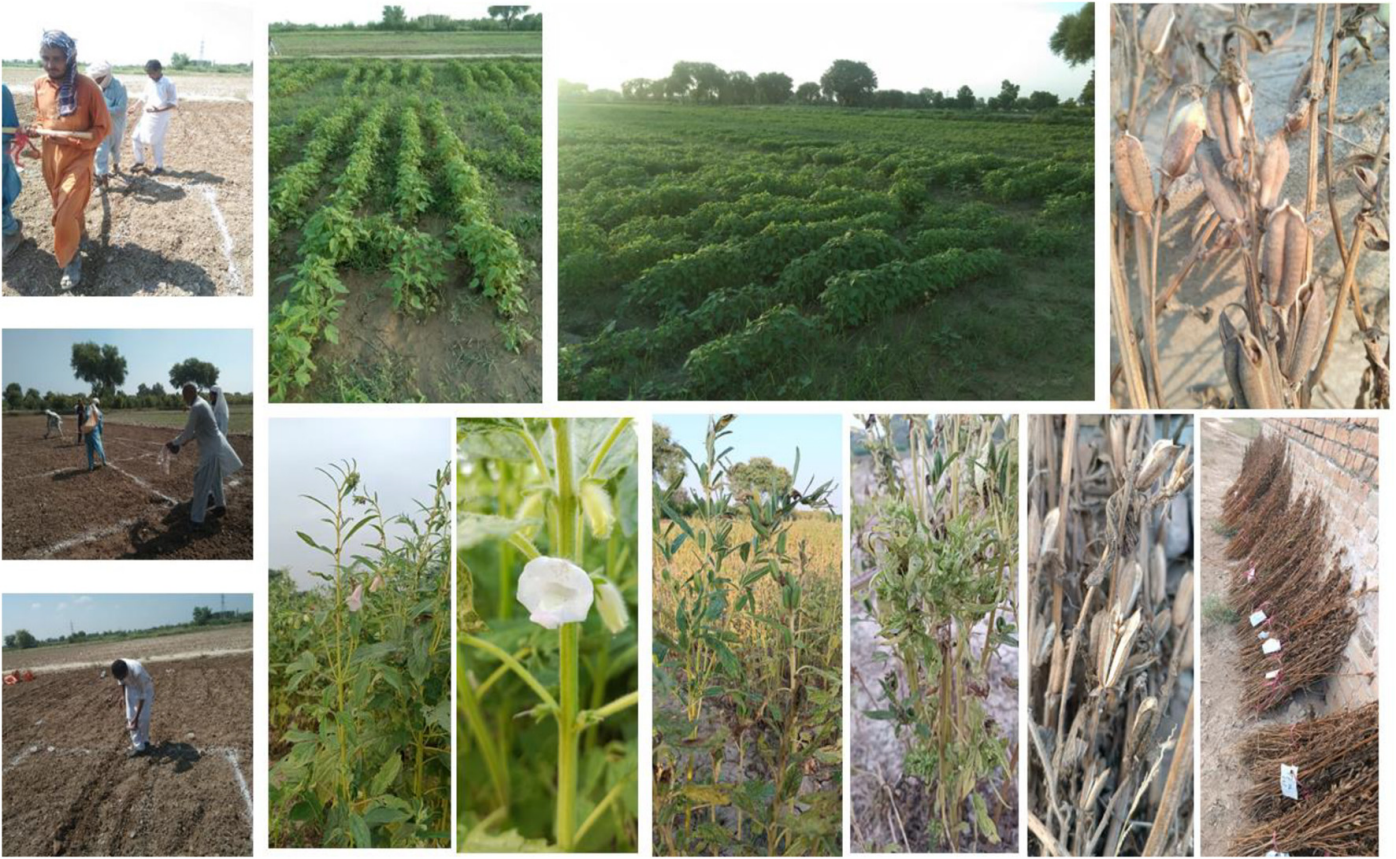
Experimental layout, crop sowing, and different stages of sesame crop in field experiment.

Field experiment was conducted at a Pakistan agriculture research station (PARC), Dera Ismail Khan, from June to November, 2023 (**Figure 1**). Seeds of six sesame verities, namely, TS-5(V1), TH-6 (V2), Til-18 (V3), Niab Millenium (V4), Niab Pearl (V5), and NS 16(V6), were collected from different research institutes. Seeds of V1 and V2 were obtained from the oilseed program of the National Agriculture Research Centre (NARC) Islamabad, Pakistan; V3 seeds were obtained from Ayub Agriculture Research Institute Faisalabad, Pakistan (AARI), while the rest of the three varieties, V3–V5, were kindly provided by the Nuclear Institute for Agriculture and Biology (NIAB), Pakistan. All the seeds were surface sterilized with 0.5% sodium hypochlorite solution followed by washing with distilled water three times. Five different treatments of SeNPs, 10PPM (T1), 20PPM (T2), 30PPM (T3), 40PPM (T4), and 50PPM (T5), were made, and seeds were divided into three groups of treatments. Group 1 had only seed pretreatment at different concentrations (T1–T5) of SeNPs, which were soaked with SeNP solution for 3 h before sowing, Group 2 was treated only with foliar spray of SeNPs (no seed pretreatment), and Group 3 had both seed pretreatment along with foliar spray at different plant life cycle condition. Foliar spray was done at five leaf stages, at maturity, flowering stage, and at fruit formation. Well-drained field with sandy loam texture soil was selected for the crop with pH of 7.3. Crops were sown in rows in triplicates with row to row distance of 20 cm and block to block distance of 3 ft. A crop was sown with hand drill to get more précised and controlled sowing. All the varieties were sown at their appropriate time of sowing, as some of them are early and some are late sowing varieties, with about a maximum of 15 days difference. Groups 2 and 3 were sprayed with approximately 200 mL of different concentrations of SeNPs at different maturity conditions. Leaf sampling for biochemical and physiological analyses and morphometric analysis was done at maturity. At full dry down stage, the crop was harvested and placed in the field upside down to become totally dry. After drying, threshing was done with hands to get reduced loss from threshing. Seed samples were packed in sterilized, air tight zipper bags and were stored at 4°CC until further analysis.

### 2.4 Morphometric and agronomical analysis

Morphological and agronomical traits data like plant height, number of capsules/plant, number of seeds/capsule, and 1,000 seed weight were noted. To get 1,000 seed weight, batches of bulk seeds were taken, and three batches of 1,000 seeds were counted with seed counter, weighed on electrical balance, and mean weight calculated.

### 2.5 Chlorophyll content

In order to check chlorophyll a, b and total chlorophyll, the method established by (Kamble et al., 2015) was used. Fresh leaf material of approximately 1 g was ground, combined with 20 mL of 80% acetone and 0.5 g of magnesium carbonate (MgCO_3_), and the absorbance was measured at 645 nm and 663 nm using a spectrophotometer, with 80% acetone solution serving as blank. Chlorophyll a, b and total chlorophyll content in mg g^−1^ was estimated by using the following formula:


Chlorophyll a=12.7(A663)−2.69(A645)X V/1000×W

Chlorophyll b=22.9(A645)–4.68(A663)X V/1000×W

Total chlorophyll=20.2(A645)+8.02(A663) X V/1000×W

where

W = fresh weight of the sample taken for analysis

V = final volume of the chlorophyll extract in 80% acetone

A = absorbance

### 2.6 Sample preparation for enzyme activity

All the leaf samples in triplicates were homogenized in 5 mL of 50 mM sodium phosphate buffer with pH of 7.8. The resulting solution was centrifuged at 12,000 rpm for 15 min at 4°CC. The supernatant was taken and stored for further enzymatic analysis.

### 2.7 Superoxide dismutase, peroxidase, and catalase activity of leaf samples

Superoxide dismutase (SOD) activity was measured according to the methods developed by (Giannopolitis and Ries, 1977), 3 mL of mixed solution containing 63 mM nitro blue tetrazolium chloride, 13 mM of methionine, 50 mM of phosphate buffer, 1.4 mM of riboflavin and 50 mL of the enzyme extract was incubated for 15 minutes and absorbance was checked at 560 nm. Peroxidase (POD) activity was measured for all the control and treated samples by following the method of Denaxa et al., 2020 by using Guaiacol as substrate and measuring the absorbance at 470 nm. To determine catalase (CAT) activity, 3 mL of the reaction mixture (50 mM PBS, 15 mM H_2_O_2_) was mixed with 50 mL of enzyme extract. The reaction was started by the addition of 100 µL of extract; an absorption decrease in H_2_O_2_ was measured at 240 nm (Cakmak and Marschner, 1992).

### 2.8 Ascrobate peroxidase, glutathione peroxidase activity, and lipid peroxidation

Ascrobate peroxidase (APX) activity was measured according to the method developed by Nakano and Asada, 1981. One gram of each leaf sample was ground in an extraction solution containing PBS (50 mM), ascorbate (2 mM), and EDTA (5mM), milled vigorously to achieve higher extraction yield and centrifuged at 13,000 rpm for 15 min. A reduction in absorbance of ascorbate was used as the indication of APX activity at 290 nm. A single unit of APX activity was determined as the enzyme necessary to catalyze the oxidation of 1 mmol of ascorbate per minute. The glutathione peroxidase activity of sesame leaf samples was measured according to the method developed by Nakano et al (Nakano and Asada, 1981). Lipid peroxidation is an important parameter used to check the integrity and stability of biological membrane. The integrity of biological membranes was checked by measuring the amount of malondialdehyde (MDA) produced by thiobutyric acid assay. Approximately 1 g of the plant material was extracted with 5 mL of 1% trichloroacetic acid, centrifuged at 1,300 rpm, for 15 min, and 1 mL of supernatant was added to 4 mL of 20% thiobutyric acid. The solution mixture was heated at a temperature of 95°CC for 30 min, and the reaction was stopped by chilling it on ice for 30 min. The absorbance of the solution was measured at 450 nm, 532 nm, and 600 nm. MDA content was calculated by using the following formula (Hodges et al., 1999):


MDA=6.45×(A532–A600)–0.56×A 450

### 2.9 Sesame seed extract for chemical antioxidant analysis

The antioxidant compounds in sesame seed samples were extracted and subsequently used for different antioxidant analysis. Sesame seed samples were ground in a coffee blender to a fine powder, and 0.1 mg of the powdered sample was mixed with 1,000 µL of methanol, followed by vortexing, sonication, and centrifugation at 13,000 rpm, 15 min each for three times in total. Supernatant was collected and was stored at −20°CC until further antioxidant analysis (Peng et al., 2021).

### 2.10 Trolox equivalent antioxidant activity

In order to measure the Trolox equivalent antioxidant activity (TEAC) activity, ABTS^•+^ was generated by reacting 7 mM ABTS stock solution in water with 2.45 mM of potassium persulfate and keeping it for 16 h in darkness. The ABTS^•+^ solution was then diluted with absolute ethanol until the absorption was reached at 0.70 ± 0.02 at 734 nm. Briefly, 500 µL of ABTS^•+^ solution was mixed with 20 µL of sesame seed extract; the solution was incubated for 6 min at room temperature followed by reading the absorbance at 734 nm. Different concentrations of Trolox (6-hydroxy-2,5,7,8-tetramethylchroman-2-carboxylic acid) were used to generate standard curve (Peng et al., 2021).

### 2.11 2,2-Diphenyl-1-picrylhydrazyl (DPPH) activity

2,2-Diphenyl-1-picrylhydrazyl (DPPH) solution (0.1 M) was used to check the radical scavenging activity of the sesame seed samples by modifying the method of Sokamte et al., 2019. Briefly, approximately 25 µL of seed extract of each sample was mixed with 275 µL of DPPH solution and incubated at 25°CC for 30 min in darkness, and absorbance was noted at 517 nm. Results were expressed in percent radical scavenging activity.

### 2.12 Reducing power assay

Approximately 10 µL of sesame seed extract of each sample was mixed with 25 µL of 0.2 M PBS solution with adjusted pH of 6.6 followed by the addition of 25 µL of C_6_N_6_FeK_3_ incubated for 20 min at 25°CC. The reaction was stopped by the addition of 25 µL of trichloroacetic acid (TCA), followed by the addition of 8.5 µL of iron (III) chloride and 85 µL of distilled water. The standard solution of different concentrations of ascorbic acid was also made to generate the standard curve. Absorbance of all the samples and standard was measured at 750 nm, and the results were expressed as ascorbic acid equivalent per gram (AAE/g) (Ferreira et al., 2007).

### 2.13 3-Ethylbenzothiazoline-6-sulfonic acid activity

In order to check the percent radical scavenging activity of sesame seed extract samples, previously generated ABTS^•+^ radical was used. For the assay, 100 µL of seed extract was mixed with 2.9 mL of ABTS^•+^ solution, while absolute ethanol served as control. All the samples were incubated in darkness at room temperature or 6 min, and absorbance was read at 734 nm with a Spectramax 250 microplate reader (Ferreira et al., 2007). Percent inhibition activity was calculated by using the following formula:


$$\text{ABTS Percent inhibition}=\frac{{\text{A}}_{\text{c}}-{\text{A}}_{\text{S}}}{{\text{A}}_{\text{C}}}\times 100$$

where

Ac = absorbance of control

As = absorbance of the sample

### 2.14 Total phenolic content

The total phenolic content of sesame seed samples was measured by Folin–Ciocalteu reagent method (Vallverdú-Queralt et al., 2014). A volume of 25 µL of Folin–Ciocalteu reagent was mixed with 25 µL of sesame seed extract samples added with 200 µL of deionized water. The solution was incubated at 25°CC for 5 min, followed by the addition of 25 µL of 10 sodium carbonate solution, and incubated for 60 min in darkness. The absorbance of the sample solution was read at 765 nm. Different known concentrations of gallic acid were used for the generation of standard curve, and total phenolic content (TPC) was given as gallic acid equivalent (GAE/g of fresh weight).

### 2.15 Oil yield of sesame treated with SeNPs

In order to get sesame oil, in 1 g of sesame seed powder, 5 mL of hexanes was added. The mixture was vortexed and placed on a magnetic stirrer at 38°CC for 20 min. The resulting solution was centrifuged at 13,000 rpm for 15 min, and the supernatant was dried with nitrogen to get oil fraction. The resulting oil was centrifuged to remove any debris and plant material. The oil obtained from 1 g of sesame seed powder was weighed, and the oil percentage was calculated by using the following formula:


$$\text{Oil percentage}=\frac{\text{Weight of oil}}{\text{Weight of sample}}\times 100$$

### 2.16 Triacylglycerol analysis of sesame oil samples

All the oil samples were analyzed for triacylglycerols (TAGs) by Liquid chromatography mass spectrometry (LCMS) method, samples were diluted 1 million times with n-butanol containing 0.1 µg of tripentadecanoin (TG) as an internal standard and transferred to LC vials. To obtain the TAG profile, a 5-µL volume of diluted oil sample was injected into an ultra-performance liquid chromatography (UPLC, Waters) system and separated using a BEH C8 2.1 × 50 mm, 1.7 µm particle size column. The mobile phase consisted of two components: A, comprising 60:40 ratio of water to acetonitrile (v/v) with 10 mmol/L of ammonium formate and 0.1% formic acid, and B, comprising methanol with 10 mmol/L of ammonium formate and 0.1% formic acid. The separation was conducted at 60°C for a duration of 10 min. The eluent was introduced into Xevo-G2-S QTOF, for ionization and MS analysis. Ammonium adducts of TAGs were detected at positive mode with energy of 30 V and 3 kV of cone and capillary voltage, respectively, for ESI. The temperature for source and desolvation were set at 120°CC and 350°CC, respectively. Nitrogen was used both as desolvation and cone gas at 50 L/h and 600 L/h, respectively, with argon used as collision gas. In order to get accurate mass measurement, calibration was done using sodium formate solution with m/z range of 50–1,500 along with intermittent injection of LockSpray leucine enkephalin with ([M+H]^+^ = *m/z* 556.2771). Accurate mass measurement was done for every TAG detected by appropriate database, elemental composition, and tandem mass spectrometry (MS/MS) fragmentation with collision energies ranging from 10 eV to 60 eV. The concentration of all TAG species was determined by calculating the relative abundance in all samples (Peng et al., 2021).

### 2.17 Data visualization and chemometric analysis of major TAG biomarkers

A multivariate data matrix was created by processing mass spectrum data and mass chromatograms using Waters’ MarkerLynx™ software. Matrix was then processed with SIMCA-P^+TM^ software (Umetrics) and pareto scaled. To define the correlations between samples and create a model for the data matrix, unsupervised principal component analysis (PCA) and O2PLS were performed. The loading plot of PCA was checked for the TAG biomarkers contributing to the separation of untreated (UT) and treated samples. As we obtained best results in Group 3 (seed + foliar treatments), in order to avoid confusions, graphical results of antioxidants are only presented for Group 3 with three best treatment concentration in Group 3 compared with untreated groups.

### 2.18 Statistical analysis:

Statistical analysis was performed with MS Excel program to get means and standard deviation of the triplicate data, while MINITAB-19 software was used to compare means of all the control and treated groups. Clustered polar heatmap, PCA, and correlation of all the TAGs were analyzed with OriginLab software. In order to check whether the treatment or variety is contributing to the differences between treated and untreated groups, the generalized linear model for analysis of variance (ANOVA) was also applied with two-way factorial design to check the level of significance between leading factors (treatment, variety, and treatment × variety) and their potential interactions.

## 3 Results

### 3.1 Characterization of SeNPs

**Figure 2:**
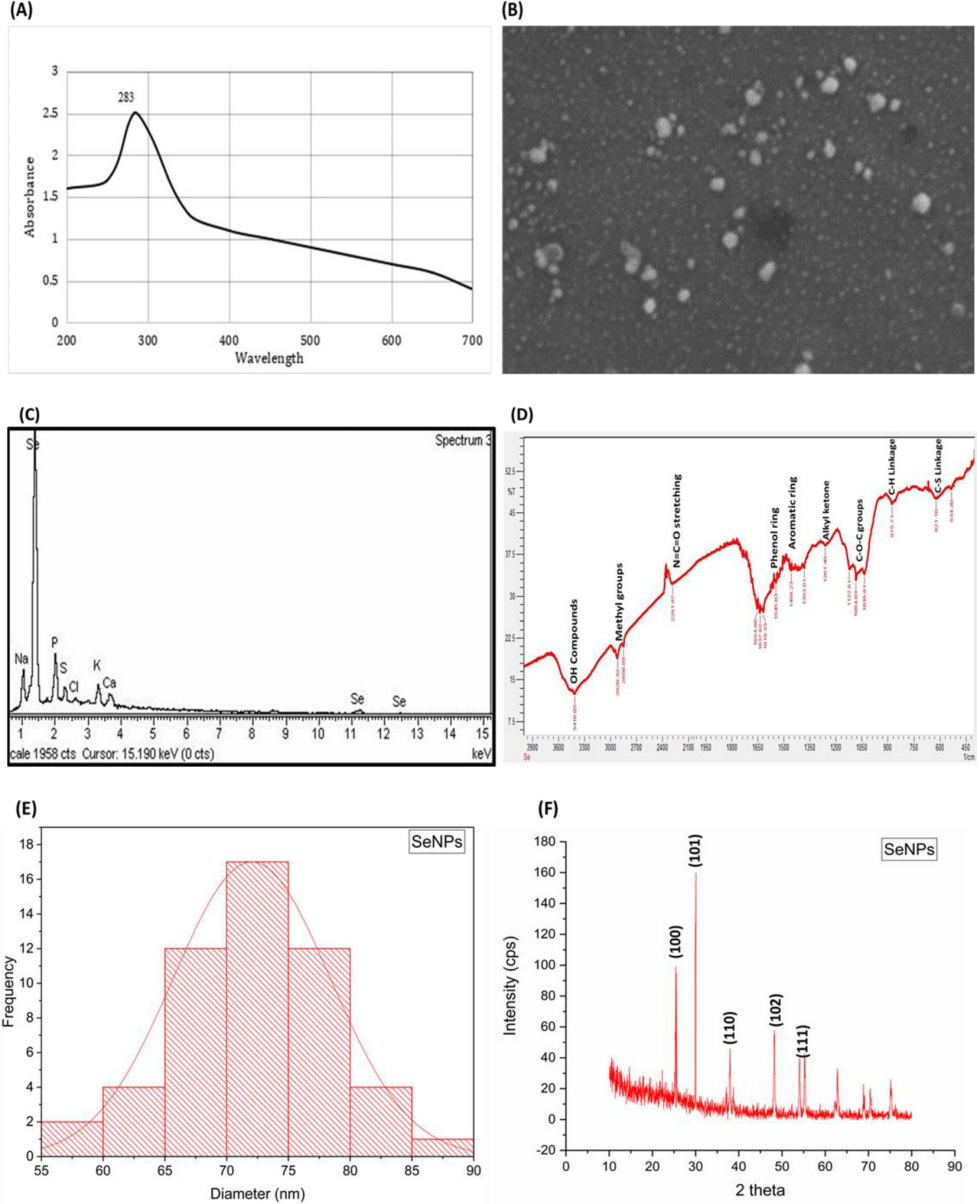
Characterization of garlic extract mediated selenium nanoparticles. **(A)** UV spectroscopy of SeNPs showing absorption peak at 283 **(B)** Scanning electron spectroscopy of SeNPs, showing nanoparticles with spherical shape. **(C)** Energy dispersive X-ray (EDX) with major peak and abundance of selenium. **(D)** Fourier transform infrared spectroscopy (FTIR) with major peaks of functional groups present on the surface of SeNPs used to reduce the selenium salt, coming from garlic extract. **(E)** Histogram showing the average size of SeNPs, showing major peak at 70–75 nm; SEM images of SeNPs were subjected to ImageJ software for average size calculation plotting histograms in Originpro and **(F)** X-ray diffraction pattern of SeNPs.

The synthesis of nanoparticles using plant extracts has contributed immensely to the field of agriculture along with sustainable production and environmental protection being the additional advantages. Medicinal plants contain a wide range of primary and secondary metabolites, which are responsible for the reduction in salts to their respective nanoparticles (Kuppusamy et al., 2016). Garlic (*Allium sativum*) is abundant in diverse bioactive compounds like allicin, diallyl sulfide, diallyl disulfide, alliin, S-allyl-cysteine, and number of flavonoids and sulfur-containing compounds, contributing to its antibacterial, antifungal, antimicrobial, antioxidant, cardiovascular protectant, anticancer, and hepatoprotective activities (Shang et al., 2019). Due to this wide range of bioactive compounds and medicinal properties, garlic was selected for the synthesis of SeNPs. The confirmation of SeNPs was indicated by a clear shift from colorless to the brick red color formation, as we gradually added garlic extract and heated the solution, suggesting the interaction of garlic bioactive compounds with selenite salt, reducing and changing them to SeNPs. Garlic extract acts as both reducing and stabilizing agent to generate environment-friendly SeNPs. Furthermore, UV analysis of SeNPs showed an absorption peak at 283 nm, further confirming the synthesis of SeNPs. Peaks at 283 and 310 showed the surface plasmon resonance of characteristics of garlic extract-mediated SeNPs. These major peaks ranging from 283 to 310 can be attributed to the spherical structure of selenium nanoparticles. Structural analysis and morphology of SeNPs were analyzed with scanning electron microscopy (SEM). SEM analysis of SeNPs showed that synthesized particles are cylindrical, spherical, or rectangular in shape. However, it was also noted that some of the SeNPs were also in irregular forms. Energy dispersive X-ray (EDX) showed the elemental composition of the SeNPs, showing the purity and abundance of selenium along with some other chemical entities like carbon, nitrogen, and sulfur, which usually surround and contribute to the stability of nanoparticles. Fourie transform infrared spectroscopy showed the major functional groups contributing to the reduction, formation, and stabilization of SeNPs. The peak at 621.10 suggested the presence of C-S linkage, while at 875.71, it suggested the presence of C–H linkages coming from the garlic extract. The major peaks in spectra 1,545.03 and 1,458.23 suggested the presence of phenol and aromatic rings, respectively, contributing to the reduction and stabilization of SeNPs. Furthermore, many other major groups like O–H stretching, methyl groups, N=C=O stretching, and C–O–C groups were major contributors for the synthesis of SeNPs. The presence of such diversity of these functional groups suggests the diverse phytochemical profile of garlic, contributing to the surface morphology of SeNPs (**Figure 2**).

### 3.2 Morphometric and agronomic characters of sesame affected by SeNPs

The primary impact of SeNPs was measured by evaluating the morphological and agronomical data of plants at maturity. Plant height, number of capsules/plant, number of seeds per capsule, and 1,000 seed weight all were highly significantly different (*p ≤* 0.05). Plant height was mainly affected by treatment of SeNPs, and highest values were noted at T5 (50ppm) of SeNPs with application method of seed + foliar. Highest plant height (133.55 cm) was noted in V3 treated with 50 ppm (T5) of SeNPs. It was also noted that the number of capsules per plant were also significantly affected by SeNP treatment, as the highest values (204) were noted again in V3 at T5 treatment. Furthermore, for the number of seed per capsule, T4 (40 ppm SeNPs) was shown to be the most effective, as the highest values of 74, 71.5, 75, 71, and 69.5 were noted in all the varieties, except V5 in which the highest values were noted at T5. Moreover, 1,000 seed weight showed a dose-dependent response with increasing trend from control to T5, as the highest values were noted at T5 in all the varieties except V3 and V6 where the highest percent change, 5% and 13%, was noted at T4 treatment. Highest percent increase (175%) in 1,000 seed weight was noted at T5 in V4, followed by V2 (70%), V1 (28.5%), and V5 (25.5%) (**Table 1**). It was evident from the data that SeNPs showed a prominent improvement in agronomical and morphological characters, leading to the improved growth parameters as compared to control plants. But at the same time, it was also found that all the varieties behaved differently to increasing concentrations of SeNPs, suggesting the genotypic response to SeNPs.

**Table 1:** Morphometric analysis and oil yield of sesame treated with selenium nanoparticles.

Variety	Treatment	Plant Height	No. of Capsules	No. of Seeds	1,000 Seed weight	Oil %
V1	0 ppm	100.95 ± 3.66 ef	127 ± 4.24 j	59.5 ± 4.95 ef	4.66 ± 0.0566 cd	49.1± 1.12 i
	30 ppm	109.55 ± 2.33 de	136 ± 2.83 gh	64 ± 4.24 de	4.735 ± 0.0778 cd	51± 1.25 de
	40 ppm	112.9 ± 6.08 cd	145 ± 4.24 ef	74 ± 4.24 b	4.855 ± 0.0354 c	53.7± 1.55 de
	50 ppm	125.95 ± 7.71 bc	135 ± 2.83 ij	72 ± 2.83 bc	4.945 ± 0.0495 c	50.1± 1.63 c
V2	0 ppm	102.6 ± 5.09 ef	100 ± 1.41 l	53 ± 5.66 f	4.335 ± 0.0354 e	51.1± 1.22 gh
	30 ppm	99.65 ± 3.32 f	105.5 ± 4.95 k	61.5 ± 6.36 e	4.465 ± 0.0354 de	50.3± 1.66 e
	40 ppm	117.35 ± 8.98 c	114 ± 4.24 kl	71.5 ± 3.54 bc	4.885 ± 0.1061 c	55.1± 1.98 d
	50 ppm	123.6 ± 7.64 bc	128 ± 1.41 j	70 ± 2.83 c	5.035 ± 0.0636 bc	53.9± 1.35 d
V3	0 ppm	110 ± 3.39 d	188.5 ± 7.78 b	72.5 ± 4.95 bc	5.095 ± 0.0212 b	57.1± 1.33 fg
	30 ppm	123.1 ± 6.36 bc	204 ± 7.07 ab	78 ± 5.66 a	5.155 ± 0.0212 b	53.1± 2.34 c
	40 ppm	130 ± 6.93 ab	210.5 ± 9.19 a	75 ± 4.24 ab	5.145 ± 0.0919 b	56.9± 2.61 a
	50 ppm	133.55 ± 8.13 a	204 ± 5.66 ab	71.5 ± 4.95 bc	5.045 ± 0.1202 bc	53.8± 2.94 b
V4	0 ppm	108.45 ± 5.3 de	142 ± 7.07 fg	54.5 ± 4.95 f	4.43 ± 0.0707 de	55.8± 1.94 h
	30 ppm	105.6 ± 4.67 e	153 ± 4.24 d	60 ± 8.49 e	4.715 ± 0.0636 cd	54.7± 1.68 e
	40 ppm	116.75 ± 3.61 c	160 ± 4.24 cd	71 ± 5.66 bc	4.75 ± 0.0424 c	59.9± 1.55 e
	50 ppm	122.3 ± 5.8 bc	156 ± 5.66 d	65.5 ± 4.95 d	6.18 ± 2.21 a	53.9± 2.31 e
V5	0 ppm	109.7 ± 8.63 de	156.5 ± 2.12 d	63.5 ± 6.36 de	4.835 ± 0.0354 c	57.3± 2.44 gh
	30 ppm	110.9 ± 2.83 d	173.5 ± 7.78 bc	68.5 ± 7.78 c	4.905 ± 0.0354 c	54.8± 2.88 b
	40 ppm	113.45 ± 8.56 cd	176 ± 8.49 bc	58 ± 4.24 ef	4.955 ± 0.0495 c	57.19± 2.91 c
	50 ppm	122.9 ± 7.92 bc	167 ± 2.83 c	65.5 ± 2.12 d	5.09 ± 0.0707 b	55.8± 2.33 c
V6	0 ppm	100.7 ± 3.54 ef	115.5 ± 4.95 kl	62 ± 1.41 e	4.885 ± 0.0354 c	58.9± 2.44 h
	30 ppm	106.6 ± 4.24 e	134 ± 5.66 ij	69.5 ± 6.36 c	5.42 ± 0.622 ab	54.1± 2.55 d
	40 ppm	110.25 ± 2.76 d	136 ± 2.83 gh	69.5 ± 2.12 c	5.015 ± 0.0354 bc	57.7± 2.67 d
	50 ppm	113.4 ± 3.96 cd	138.5 ± 7.78 gh	69 ± 4.24 c	4.865 ± 0.0778 c	56.3± 1.88 d

All the data were obtained at maturity; values are shown in means of triplicates.

Means are shown from triplicate values. UT, untreated; T, treatment. Means followed by different letters in the same column shows significant difference (p ≤ 0.05).

### 3.3 Photosynthetic pigments

**Figure 3:**
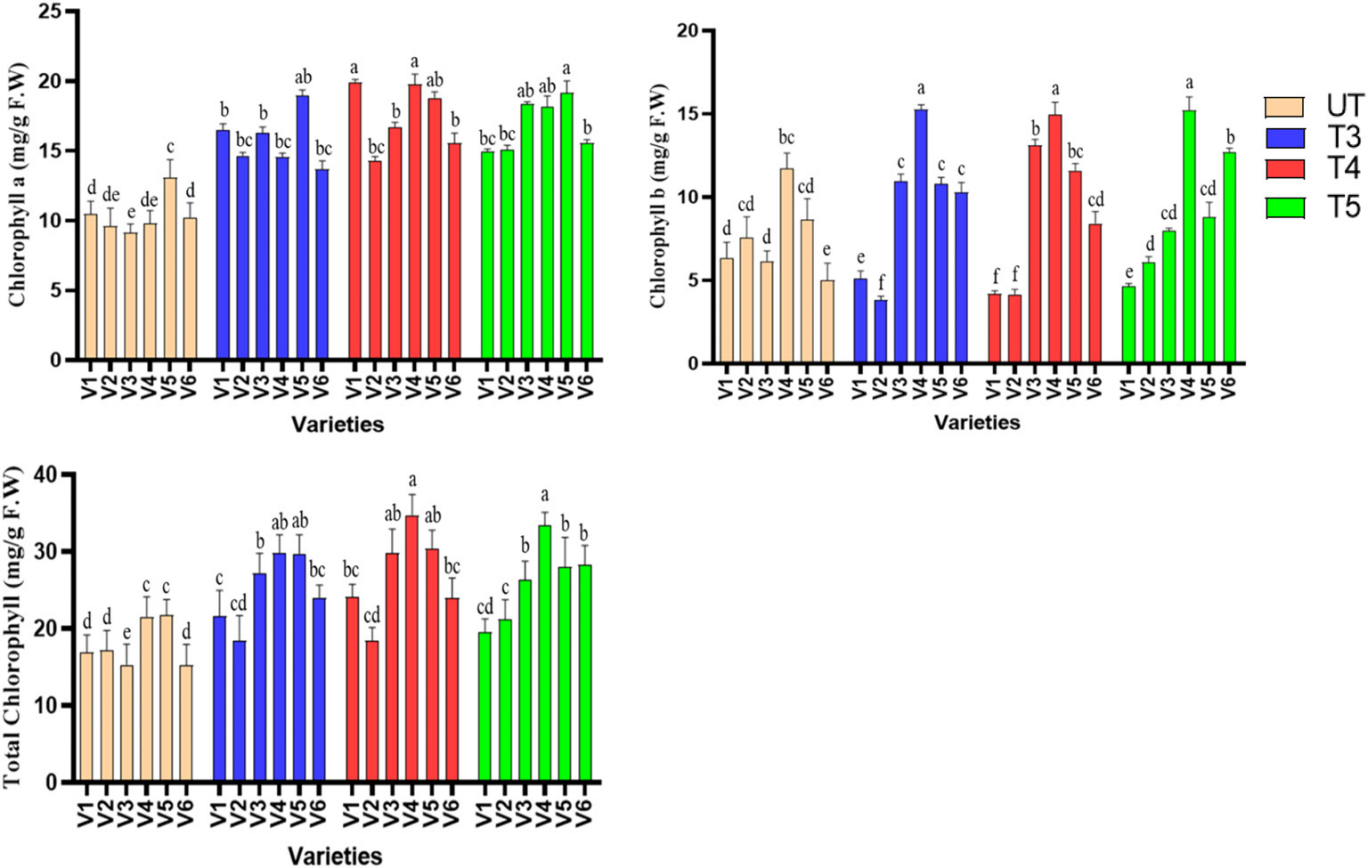
Effect of seed + foliar applications of plant-mediated selenium nanoparticles on chlorophyll a, chlorophyll b, and total chlorophyll content of sesame. Small letters show the results of statistical analysis, as DMRT was performed to check the significant differences between the means. Same letter indicates no significant difference, while different letters means significant difference.

In order to check the effect of SeNPs chlorophyll quantification was performed. Levels of chlorophyll a, b and total chlorophyll were measured spectrophotometrically. It was found that SeNPs has improved the total chlorophyll content in sesame, as the highest percent increase (94.84%) was found in V3, at 40 ppm of SeNPs, followed by 61% in V4, 57% in V6, 40% in V5, and 7% in V2 when compared to untreated plants. Furthermore, 40 ppm (T4) treatment was shown to be most effective in improving total chlorophyll content, as it increased from control to T4 and decreased again at T5, suggesting that 50 ppm treatments are somehow exerting a stress to the plants resulting in decreased total chlorophyll production. Similar results were obtained for chlorophyll A content, as the highest percent increase was found at T4 treatments in all the varieties, but the highest percent increase was noted in V1 (90%) followed by V3 (82%) (**Figure 3**).

Similarly, chlorophyll a and b contents were also significantly improved (*p ≤* 0.05) when compared to untreated plants. The highest percent increase (101%) was found in V4 at T4 treatments followed by 90% in V1 and 82% in v3. As far as the chlorophyll a content is concerned, T4 treatments were found to have been most effective, as it improved chl a content significantly in all the varieties, followed by a reduction trend at T5. In contrast, chlorophyll b has shown almost similar improvement percentage at T3 and T4 treatments and reduction trend at T5. It was concluded that SeNPs have the beneficial role in the synthesis and protection of photosynthetic pigments, which resulted in overall increase in chlorophyll content. T4 was found to be effective in almost all the cases, except chl b, which was shown to have been less affected by the SeNP treatments.

### 3.4 Lipid peroxidation, SOD, POD, CAT, APX and GPX activity of sesame treated with nanoselenium

Lipid peroxidation is the important parameter to determine the integrity of biological membrane. Oxidative stress causes damage to the membrane resulting in a membrane leakage and disturbed membrane functioning. Malondialdehyde content is the important chemical parameter to check the lipid peroxidation and resulting damage caused to membrane; it was found that SeNPs showed a deceasing trend with increasing concentrations till T4 treatments. When compared to untreated plants, the maximum percent decrease was noted in V3 at T5 treatments with −64% decrease in MDA content followed by 56% decrease in V2 at T4 treatments, as compared to untreated plants. Furthermore, it was also found that in all the varieties except V3, T4 treatments was found to have the most protective effect against membrane damage, and at T5 treatment, MDA content again tended to be in increasing trend, suggesting a mild toxicity caused by nanoparticles. It also suggests that the germplasm again also has the prominent role, as V3 is showing improvement in oxidative stress protection until T5 and is resistant to higher levels of SeNPs as compared to other varieties.

It was expected that low levels of SeNPs have the ability to modulate ROS levels in plants, resulting in the activation of response against stress, and at the same time, at a higher concentration, it also acts as a stress stimulator and produces higher levels of ROS. Superoxide dismutase (SOD) activity was at maturity, and it was found that varieties responded differently to the level of SeNPs in SOD activity. For example, V1 has shown the highest percent increase at T4 treatments, which was 74.4%, followed by a decrease to 58% at T5 treatments; a similar trend was followed by V3, V4, and V6. In contrast, in V2 and V5, the highest percent increase values were found at T5 treatments with 55.50% in V2 and 29.32% in V5 as compared to untreated plants, again suggesting an interaction of germplasm with treatments. However, all the treatments of SeNPs have improved the SOD activity when compared to untreated plants, as there was a significant difference at p ≤ 0.05 when means were compared.

The peroxidase activity of sesame leaf samples was also significantly affected by SeNPs, showing the antioxidative properties of SeNPs. The highest percent increase (43%) in POD activity was noted at T5 treatment in V5 when compared to the untreated group, as it showed increasing trend from T3–T5. In contrast, V3 showed different responses as compared to other parameters, as it displayed highest values at T4 (37.56%) and reduction in POD content at T5 treatment (34.48%), as a similar trend was followed by V4 and V6. Compared to other varieties, V6 has shown little variation in POD content, as 28% increased POD activity was found at T4 treatments when compared to control groups, suggesting that although SeNPs improve POD content, it is not constant in all the varieties.

**Figure 4:**
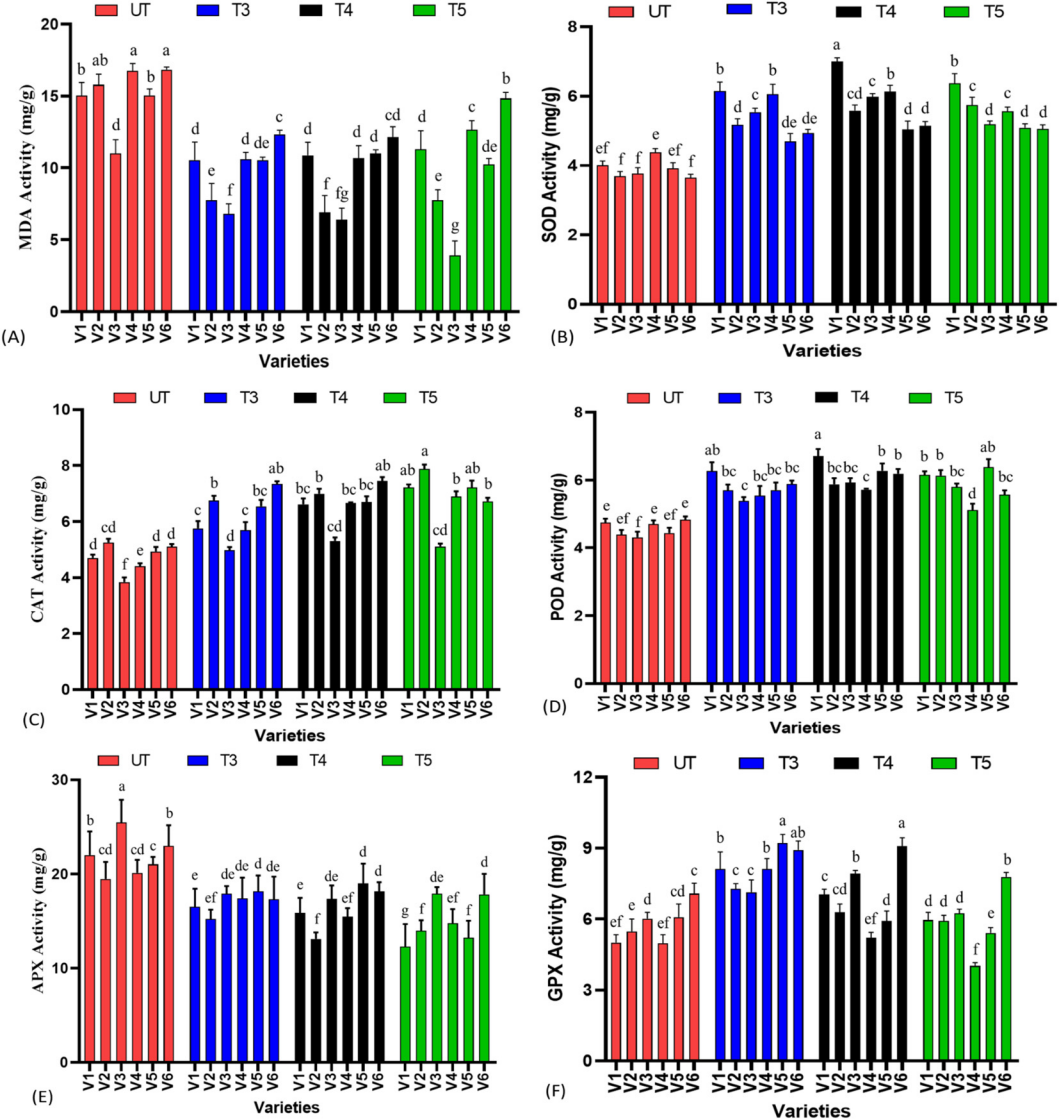
Enzymatic antioxidant analysis of sesame leaf extract treated with SeNPs, **(A)** malondialdehyde content (MDA) content, **(B)** superoxide dismutase activity, **(C)** catalase activity, **(D)** peroxidase activity, **(E)** ascorbate peroxidase (APX), and **(F)** glutathione peroxidase (GPX) activity. Small letters show the results of statistical analysis, as DMRT was performed to check the significant differences between the means. Same letter indicates no significant difference, while different letters means significant difference.

Catalase is one of the important enzymatic antioxidant arsenals present in plants to scavenge various ROS species. SeNPs had significantly improved the catalase activity sesame treated with nanoselenium. An increasing trend in CAT activity was noted from T3–T5, as the highest percent improvement was found at T5 in V1, V2, V4, and V5; in contrast, V3 and V6 showed a somewhat different response, as the highest percent increase values (38.28% and 46.16%) were found at T4 treatments, and T5 treatment again showed a decreasing trend. The highest increase in CAT activity was found in V4 (46.66%) and the lowest response was noted in V3, when compared to untreated groups (**Figure 4**).

Similar to the activity of catalase, APX and GPX are also involved in detoxification of H_2_O_2_ activities, a marker of oxidative stress in plants. Hence, any improvement in the activity of these two enzymes enables plants to cope and protect the metabolic machinery from hazardous effects of oxidative stress. It was found that GPX activity was found highest in all varieties at T3 treatments, except V3 and V4, which behaved differently, as the highest increase was found at T4 treatments in these two varieties. T3 was found to be the most effective in improving the GPX profile, and higher concentrations resulted in a gradual decrease in GPX activity. Highest percent increases of 62%, 32.84%, 53.05%, and 51.64% were found at T3 treatments in V1, V2, V4, and V5, respectively. It was also important to note that increasing concentration of SeNPs dramatically reduced the GPX content to lower than that of untreated plants, suggesting that a higher concentration, instead of improving the antioxidant profile, resulted in ROS increment; hence, a shift towards decreasing trend was noted. While in V3 and V6, highest percent increases of 31.56% and 28.24%, respectively, were noted at T4 treatments, and an abrupt decrease was noted at T5 treatments.

### 3.5 DPPH and ABTS inhibition activity of sesame seed samples

**Figure 5:**
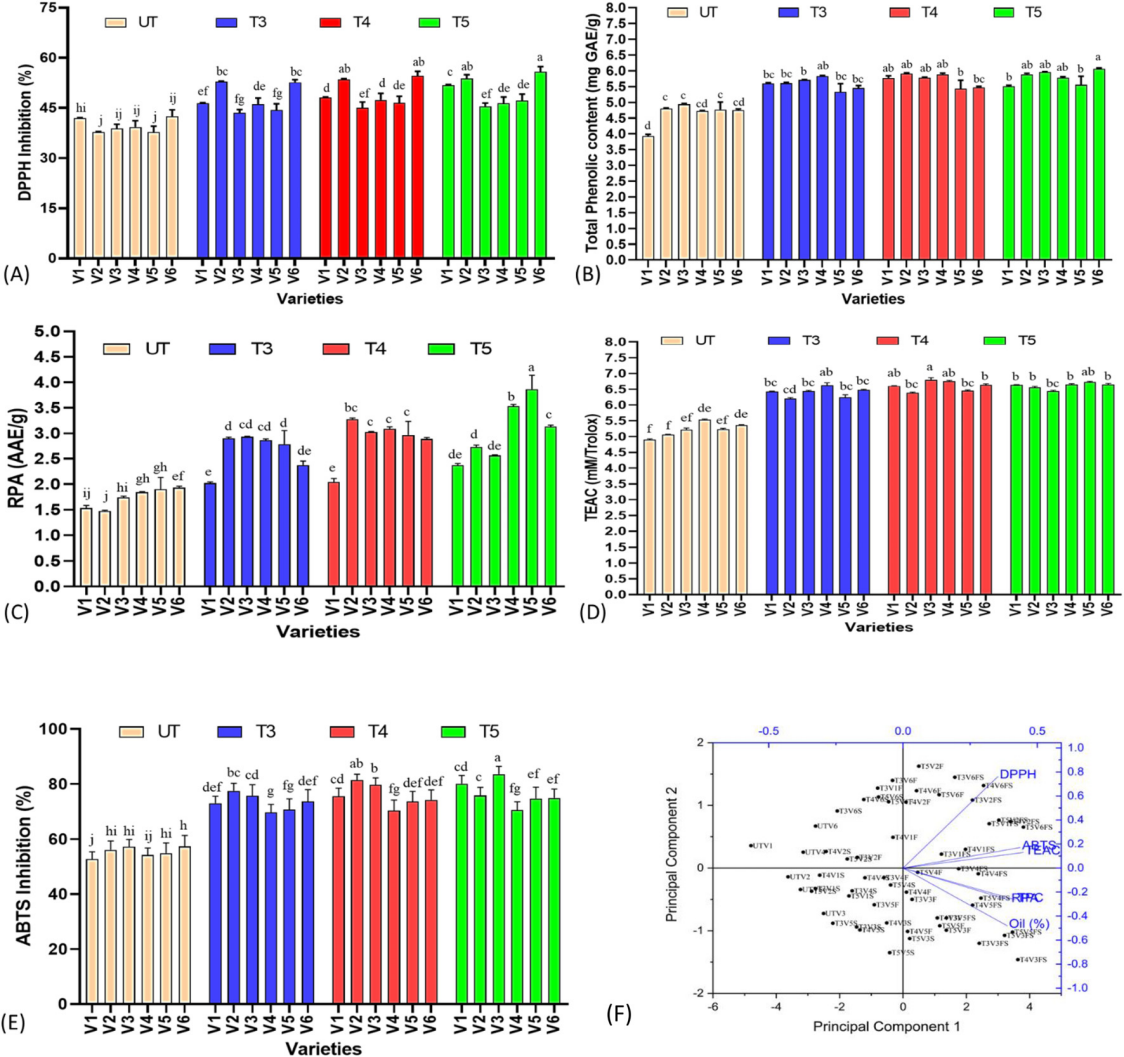
Effect of plant-mediated selenium nanoparticles on non-enzymatic antioxidant parameter of sesame seeds extract treated with SeNPs. **(A)** DPPH activity, **(B)** total phenolic content, **(C)** reducing power ability, **(D)** Trolox equivalent antioxidant activity, **(E)** ABTS inhibition activity, and **(F)** principal component analysis of non-enzymatic antioxidant parameters and seed antioxidant parameters and oil percent yield in sesame. These small letters shows the results of statistical analysis, as DMRT was performed to check the significant differences between the means. Same letter shows no significant difference, while different letters means significant difference. Small letters shows the results of statistical analysis, as DMRT was performed to check the significant differences between the means. Same letter shows no significant difference, while different letters means significant difference.

As sesame seeds are mainly utilized for food, cooking, cosmetics purposes, etc., we have further investigated the effect of SeNPs on antioxidant acuity of seed samples. DPPH assay is widely used to determine the radical scavenging activity, which is mainly attributed to phenolic acids. DPPH showed a concentration-dependent response in sesame seed samples, as lowest values were noted in the untreated group with a gradual increasing trend, with T5 having the highest percent DPPH scavenging activity in all varieties (*p ≤* 0.05), except V4, which showed highest values at T4 treatments (**Figure 5**). Highest percent increase of 42.49% was found in V2, followed by 31.50%, 25.22%, 23.63%, and 17.03% in V6, V5, V1, and V3. It was also important to note that untreated plants of some varieties like V3 already had a high percentage of DPPH radical scavenging activity; although SeNPs significantly affected the DPPH activity, percent change was found to be lower as compared to overall scavenging acidity values. In contrast to DPPH activity, ABTS has shown a somewhat different response in varieties to varying concentrations of SeNPs. It was found that T5 was the most effective treatment, as an increasing trend was noted from untreated groups to T5, except V2, which showed higher relative percent values at T4 treatments. V3 has shown 83.53% radical scavenging activity at T5 treatments, compared to 57.19% in untreated groups, followed by V1 with 80.11% activity in T5 with 52.68% in untreated plants. V2 behaved differently, as highest values were noted at T4 with percent increase of 45.22% followed by reduction with T5 treatments.

The assay known as Trolox equivalent antioxidant capacity (TEAC) quantifies a substance’s antioxidant capacity in relation to the standard Trolox. The ABTS decolorization assay is the method most frequently used to determine antioxidant capacity. It was noted that SeNP treatments also improved the TEAC activity of sesame seed extract at a maximum of 30.3% at T4 treatments in V3. Similarly, all the varieties showed the same response, and highest percent increase were noted at T4 treatments in all the varieties, as significant difference was found between untreated and treated groups (*p ≤* 0.05). It was also shown that increasing concentration of SeNPs improved TEAC activity, but at T5, a reduced activity was noted in all the varieties indicating the negative impact of a high dose of SeNPs. Furthermore, in contrast to some other antioxidant parameters, which showed highest values at T5, TEAC values were near to untreated groups at T5 treatments, suggesting an effective treatment of T4 (40 ppm).

Reducing power assay is another important antioxidant parameter based on the principle that compounds with reduction potential combined with potassium ferricyanide (Fe^3+^) to generate potassium ferrocyanide (Fe^2+^), which subsequently reacts with ferric chloride to form ferric–ferrous complex that has an absorption maximum at 700 nm, is the basis of the reducing power test method. Sesame seed extract showed a wide range of reducing power potential with lowest values of 1.47 in untreated plants of V2 to 3.86 in T5 treatments of V5. All the treated plants were significantly different (*p ≤* 0.05) from untreated plants, showing the improvement potential of SeNPs. All the sesame varieties showed a mixed response to increasing concentration of SeNPs for reducing power assay (RPA) activity. As V2 and V3 showed highest percent increase of 121.89% and 73.29%, respectively, at T4, the rest of the varieties have shown maximum percent change at T5 treatments. At T5 treatments, V5 has shown maximum increase of 103.56% followed by 91.62% by V4 and 62.10% by V6, while V2 and V3 shown a reduction in RPA activity at T5 treatments.

As sesame is rich in polyphenols, exerting an array of health benefits to plants against variety of stresses. In addition to giving protection against oxidative stress and scavenging ROS, plant phenolics are typically engaged in defense against UV radiation and hostility from infections, parasites, and predators. It was noted that SeNP treatments has a significant effect (*p ≤* 0.05) on the TPC content of sesame seeds. It was found that V1, V2, and V4 responded well at T4 treatments of SeNPs with highest percent increase in TPC content of 46.82%, 22.90%, and 24.52%, respectively, while V3 (20.68%), V5 (16.40%), and V6 (27.41%) showed highest values at T5 treatments, showing the varying response to SeNPs of all the varieties. It was also found that TPC content was highest in V3 (5.96) and V6 (6.06) when compared to all treated and untreated groups, suggesting a better candidate to be used for food purposes.

### 3.6 Percent oil yield of sesame treated with SeNPs

As sesame oil is the major product that is utilized at large scale for its various beneficial aspects, it was the major parameter to consider and to check that if the crop growth improvement has also resulted in oil yield improvement. It was found that SeNPs have significantly improved (*p ≤* 0.05) the oil yield in sesame. Highest percent increase was found in V1 at T5 SeNPs, found to be 16.47% high as compared to untreated plants. Similarly, highest oil yield values were also noted at T5 treatments in V2, V4, V5, and V6 at 9.41%, 7.98%, 12.91%, and 11.92%, respectively, when compared to untreated groups. Furthermore, it was also found that V3 has responded somewhat differently as the oil yield was highest (59.9%) in V3 at T4 treatments with percent increase of 11.54%. Therefore, it was found that T4 and T5 treatments are both effective in improving the oil yield of sesame to a considerable level depending upon the oil percent in the control ones.

### 3.7 Chemometric comparison of SeNP-treated and untreated sesame

**Figure 6:**
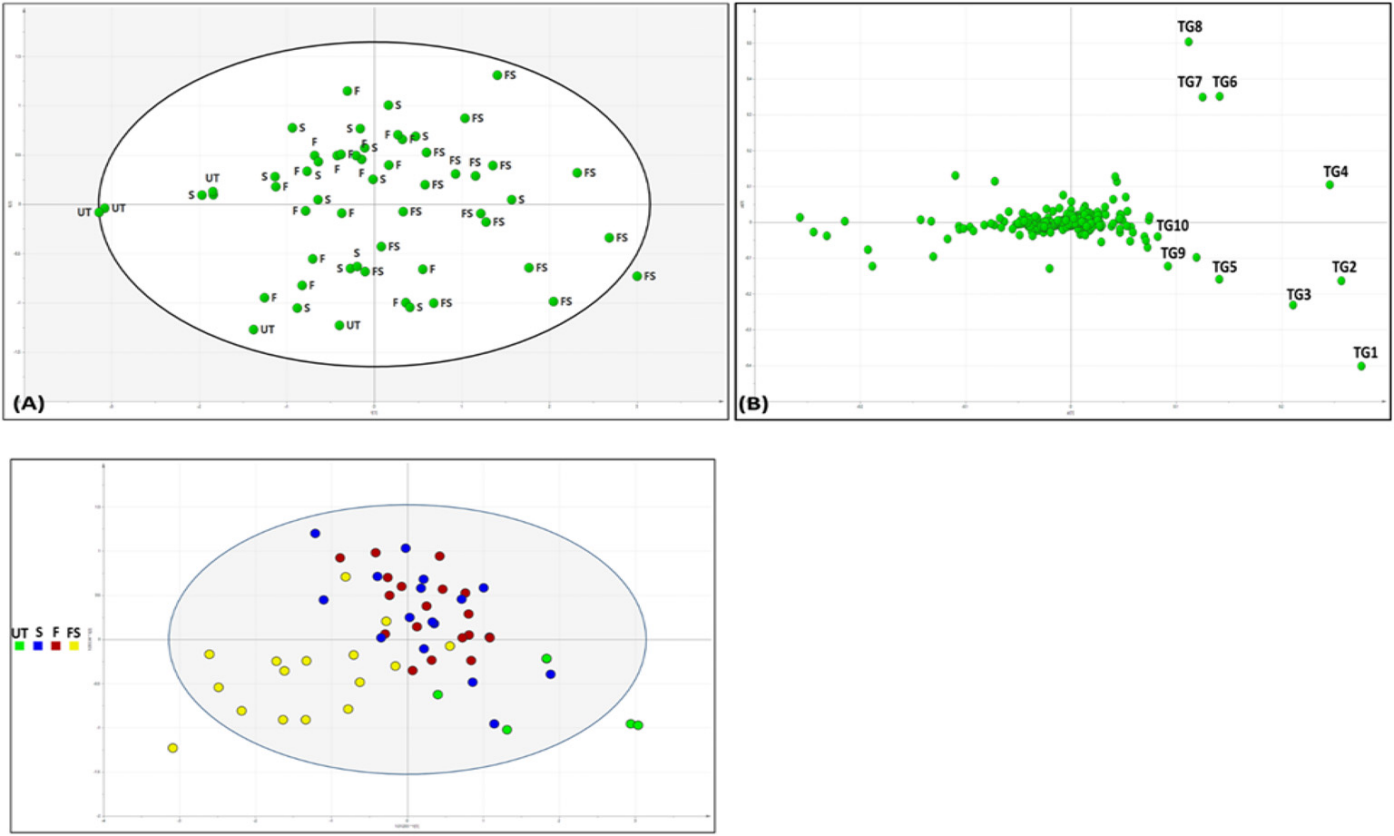
Chemometric analysis of sesame seed oil treated with varying concentrations of SeNPs on their TAG composition. **(A)** Score plot of principal component analysis showing separation of untreated and seed + foliar-treated groups. **(B)** Loading plot of the PCA model showing major TAG biomarkers (TG1–TG10) responsible for the separation of untreated and treated groups. Comparison of untreated and selenium-nanoparticle-treated samples. (c) O2PLS of sesame seed oil samples, separating samples on the basis of treatment method. Green, untreated (UT); blue, seed pretreatment (S); red, foliar spray (F); and yellow, seed + foliar treatment (FS). Suggesting seed treatment is effective, but grouping with untreated plants shows less efficacy, followed by foliar spray and FS group completely separated from UT, suggesting strong influence on TAG profile.

TAG profiling of SeNP-treated and untreated sesame seed oil samples was carried out to check if there are any differences in oil profile. It was found that although the TAG profile of treated and untreated groups was not greatly affected, the concentration of each TAGs varied significantly between treated and untreated groups groups (**Figure 6** and **Table 2**). The fatty acid composition was revealed by fragmentation by MS/MS method, generating monoacylglycerols and diacylglycerols fragments by the neutral loss of acyl groups. For example, POO (palmitic: oleic: oleic) has a major fragment of 577.5201 from the neutral loss of oleoyl group along with a minor fragment (*m/z =* 279.0945), belonging to oleic acid. In the score plot of TAG profile, it was noted that all the untreated and seed pretreated groups were clustered together followed by foliar-treated plants in the middle and all the seed + foliar samples clustered separately. It was found that the TAG profile of all the untreated groups was comparable to Group 1 (seed pretreated only), while it was different from seed + foliar group. Major TAG markers contributing to the separation of seed + foliar group from untreated ones are marked from TG1–TG10. Relative abundance of these TAGs is also given. All the TAGs identified were further clustered by heatmap clustering, resulting in three major cultures. It was noted that untreated V2, V6, and V5 groups were clustered together (clustered A) along with T3 treatment of V6, showing a low relative abundance of TAGs in these treatments. Furthermore, second culture (B) was found to be clustering T4 and T5 treatments of V1, T3 of V3 and V2, and T4 of V4, suggesting a moderate level of TAGs in these treatments. Furthermore, third culture (C) grouped T4 and T5 treatments of almost all varieties, suggesting the high levels of TAGs in this group. T4 and T5 of V3 and V6 were predominantly found in this culture, suggesting the higher response of these two varieties to SeNPs. Surprisingly, the untreated group of V3 was also clustered in the third group, suggesting that this variety already have comparatively higher levels of TAGs, as compared to other varieties. PCA analysis was also done to check the separation of untreated and treated samples for TAG composition, which confirmed that all the untreated samples of V1, V2, V4, and V5 were strongly correlated with each other and negatively correlated with T4 and T5 treatments of V3, as it showed a high abundance of TAGs. Similarly, the untreated group of V3 was also negatively correlated with untreated groups of other varieties, suggesting a higher level of TAGs in V3 variety.

**Table 2:** Major triacylglycerol species contributing to the separation of untreated and selenium-treated sesame in loading plot of PCA analysis.

ID	Formula	TG composition*	Exact calculated mass [M +NH4]	Measured m/z [M +NH4]	Deviation (PPM)	Major fragments of MS/MS (*m/z)*
**TG1**	C_57_H_104_O_6_	OOO	902.8177	902.8162	0.0015	603
**TG2**	C_55_H_102_O_6_	POO	876.8020	876.8007	0.0013	577
**TG3**	C_57_H_10_6O_6_	SOO	904.8333	904.8317	0.0016	605, 279
**TG4**	C_55_H_100_O_6_	POL	874.7864	874.7850	0.0014	601
**TG5**	C_57_H1_00_O_6_	OOL	900.8020	900.8006	0.0014	601
**TG6**	C_55_H_98_O_6_	PLL	872.7707	872.7692	0.0015	575, 279
**TG7**	C_57_H_100_O_6_	OLL	898.7864	898.7848	0.0016	601
**TG8**	C_57_H_98_O_6_	LLL	896.7707	896.7691	0.0016	599
**TG9**	C_55_H_104_O_6_	PSO	878.8177	878.8158	0.0019	605, 279
**TG10**	C_59_H_108_O_6_	OLA	930.8045	930.8457	-0.0412	526, 279

*O, oleic acid (C18:1); P, palmitic acid (C16:0); S, stearic acid (C18:0); L, linoleic acid (C18:2); A, arachidic acid (C20:0).

### 3.8 Relative abundance of major TAG markers

**Figure 7:**
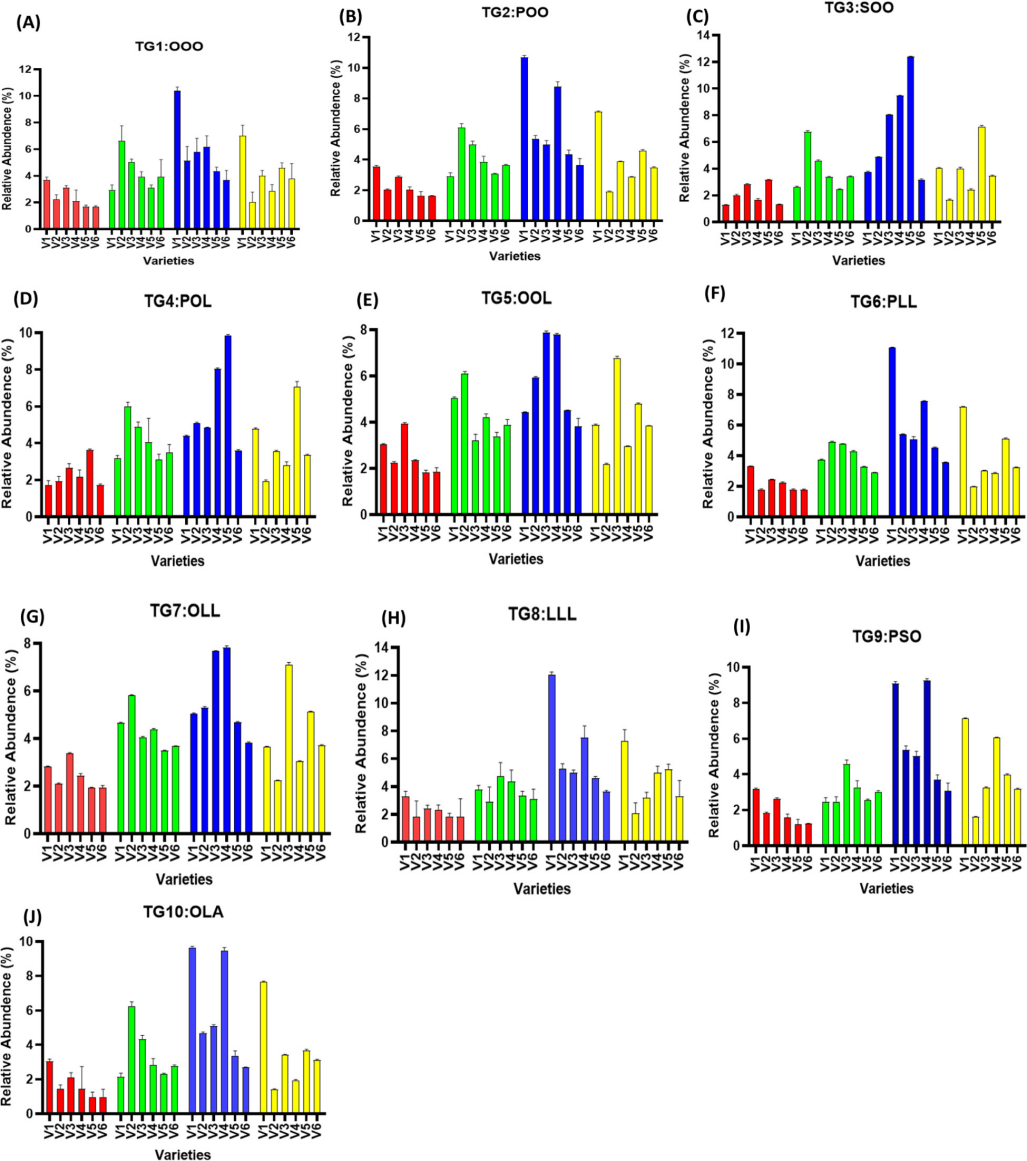
**(A–J)** Relative abundance of major TAG biomarkers (TG1–TG10) in untreated and SeNP-treated sesame seed oil samples.

**Figure 8:**
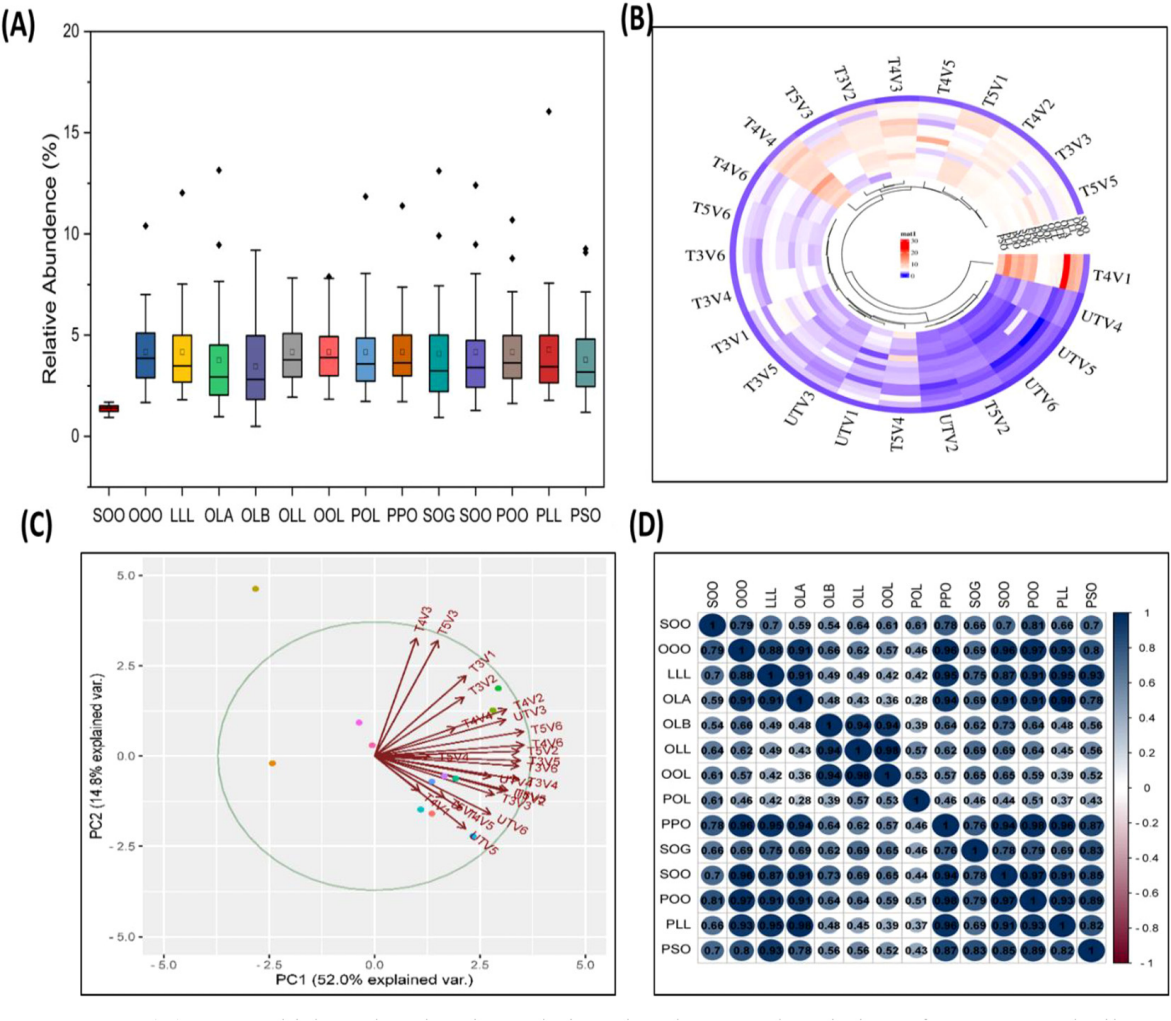
**(A)** Box–whisker plot showing relative abundance and variation of sesame seed oil triacylglycerol (14 in total) from lower (UT) to highest values (T4 and T5). **(B)** Clustered heatmap of SeNP treatments on the basis of TAG concentration, clustering almost all untreated group together and separated from T4 and T5 treatments. **(C)** PCA analysis of samples on the basis of SeNP treatments and TAG profile, suggesting a separation of untreated and treated groups. **(D)** Pearson correlation coefficient matrix with correlation value, correlating TAG species with each other.

Relative abundance of TAGs in treated and untreated groups varied significantly, as higher TAG concentration was noted in treated groups as compared to untreated ones. TG1 (OOO) has been noted to have the highest values in V1, with relative percent increase of 181.10% at T4, followed by 159.49% increase in V5. It was noted that T4 treatment had the highest percent increase in TAG content for TG2 (POO), TG4 (POL), TG6 (PLL), TG7 (OLL), TG8 (LLL), TG9 (PSO), and TG10 (OLA), when compared to untreated ones, and T5 shows a decrease in TAG content. It was also noted that TAG content seems to be variety specific, as in each time, some of the varieties had very high levels of TAGs compared to others, but the overall profile of TAG was found to be improved by SeNPs (**Figures 7**, **8**).

## 4 Treatment or variety?

As we used six different varieties with application of various concentrations of SeNPs, it was imperative to check which one is the major factor contributing to the variation among treated and untreated groups. GLM with two-factorial ANOVA proved that SeNPs have highly significant impact in the enhancement of antioxidant and oil biomarkers. Statistical analysis suggested that the number of seeds/capsule, Chl a, SOD, POD, CAT, APX, GPX, MDA, DPPH, ABTS, TEAC, TPC, RPA, and Oil % were highly significant (*p ≤* 0.001) with SeNP treatments. In some of the cases like for 1,000 seed weight, Chl b, total Chl, SOD, CAT, APX, and DPPH, varieties were shown to have significant impact (*p ≤* 0.05), but the covariation of treatment and variety was not significant, suggesting that treatment had the major impact on changing the metabolic profile of the sesame. Furthermore, significant values for varieties can be attributed to the variation found between the untreated groups of sesame varieties. For example, percent oil content varied from 49% to 54% untreated groups (V1 and V3, respectively), and TPC content [3.93 (lowest) to 4.93 (highest) mg GAE/g] between untreated groups, suggesting the differences in genetic and phenotypic ability of different sesame varieties (**Table 3**).

**Table 3:** *p*-Values obtained from generalized linear model (GLM) of ANOVA with two factorial design for treatment and varieties.

Crop growth parameters	*p-*Values		
	Treatment	Variety	Treatment × variety
Plant height	*	NS	NS
Number of capsule plant**^−1^**	*	**	NS
Number of seeds/capsule	**	**	NS
1,000 seed weight	*	*	NS
Chl a	**	NS	NS
Chl b	*	*	*
Total Chl	**	*	NS
SOD	**	*	NS
POD	**	NS	NS
CAT	**	*	NS
APX	**	*	NS
GPX	**	NS	NS
MDA	**	**	NS
Seed parameters			
DPPH	**	*	*
ABTS	**	NS	NS
TEAC	**	NS	NS
TPC	**	*	NS
RPA	**	NS	NS
Oil %	**	*	NS
TAG markers			
OOO	*	NS	NS
POO	*	NS	NS
SOO		NS	NS
POL	*	NS	NS
OOL	*	NS	NS
PLL	*	NS	NS
OOL	*	NS	NS
LLL	*	NS	NS
PSO	*	NS	NS
OLA	NS	NS	NS
Other TAGs			
SOG	*	NS	NS
SOO	NS	NS	NS
OLB	*	NS	NS
SOO	*	NS	NS

*significant (p ≤ 0.05), **highly significant (p ≤ 0.001); NS, not significant.

## 5 Discussion

The global population is projected to reach 9.6 billion by the end of 2050, necessitating a 70%–100% rise in crop productivity to adequately fulfill the demands of this growing populace (Rodrigues et al., 2017; Alabdallah et al., 2021). Nevertheless, significant yield losses are incurred due to the escalation of global warming and climate change, low yield germplasm, diminishing fertile land, excessive utilization of fertilizers and pesticides, and heightened intensity of abiotic stresses (Hasan et al., 2021a; Hasan et al., 2021b). Consequently, the decline in crop productivity presents a significant risk to global food security. Thus, it is crucial to implement suitable measures aimed at mitigating the adverse effects of not only the climate but also of hazardous chemical fertilizers, insecticides, and pesticide, which not only cause environmental pollution but also put a major economic concerns to farmers (Genc et al., 2019; Hasan et al., 2021b). Pakistan being the agricultural country, with 26% contribution to GDP, not only is the source for food but also provides employment (67.5% population is directly associated with it) and contributes to 45% of total exports of Pakistan (Azam and Shafique, 2017). As Pakistan population is increasing dramatically, at a rate of 33%, there is a dire need to increase food production by at least 40% to provide sufficient food to the population. As Pakistan is major importer of edible oil, importing 88.6% with only 11.4% of local production, by spending 4.5 billion USD, is a huge concern for our economy (Rana et al., 2022). One major concern for agriculture in Pakistan is the lack of agriculture inputs, like fertilizers, as we have only 10 production units, which results in black marketing of the fertilizers when farmers needed them the most. Therefore, there is a great need to go for alternative approaches that can improve the crop growth at low cost, and at the same time, it should not affect the environment. Here comes the twenty-first century science “Nanotechnology,” which operates with materials at nanoscale, which now became a solution of many problems with an ecofriendly solution of nanotechnology (Abdel-Aziz et al., 2019).

Sesame, belonging to family Pedaliaceae, is an important oil seed crop that is rich in oil, along with protein, carbohydrates, dietary fiber, phytosterols, tocopherols, and other natural antioxidants. These characteristics make sesame as a potential crop to contribute to the Pakistan edible oil requirements along with fulfilling the food demands of Pakistan’s increasing population. At the same time, oil seed crops and sesame are not given attention as compared to other staple crops like wheat, hence missing the potential they have for the farmers, consumers, and Pakistan’s economy. Sesame is also neglected in the research field, hence a very low number of high yielding varsities; less information about fertilizer use, irrigation, and soil preparation are available, resulting in low yield, due to which farmers restrain themselves from sowing this crop. Therefore, considering the potential of sesame for multiple purposes, this study was conducted to check the impact of plant-mediated selenium nanoparticles on crop growth performance, oil yield, fatty acid, and metabolic profile.

At low quantities, selenium is an essential element needed for the proper physiological and biochemical functioning of plants and animals, but at higher doses, it becomes hazardous and toxic. Plant-based selenium nanoparticles, or SeNPs, have emerged as one of the best antibacterial and antioxidant nanomaterials to lessen the harmful impacts of stress and along with improved crop yield (Zohra et al., 2021). It has been demonstrated that SeNPs made from plant extract exhibit exceptional biocompatibility, bioavailability, reduced toxicity, biodegradability, and environment friendliness. This is due to the presence in plant extracts of secondary metabolites of tannin sesquiterpenes, monoterpenes, phenolic acid, and cinnamic acid, which function as reducing agents and stabilize capping during the creation of SeNPs, thereby giving them their biocompatible nature (Ahmad et al., 2024). Additionally, some research showed that phytogenic SeNPs react 20 times faster than bulk selenium (Alagesan and Venugopal, 2019). Therefore, plant-mediated SeNPs were considered for the present study to improve the growth performance and oil yield of sesame.

Photosynthetic pigments, particularly chlorophyll, are pivotal in capturing light and converting it into energy for carbohydrate synthesis. Any changes in chlorophyll levels can significantly impact plant metabolism. The current study demonstrates that SeNPs have a beneficial impact on photosynthetic pigments, as evidenced by an increase in chlorophyll content with rising concentrations of SeNPs. Furthermore, foliar application of SeNPs is suggested to yield higher total chlorophyll content compared to seed priming alone (Salama, 2012). These findings align with similar results reported by previous research, conducted on *Brassica juncea*; they came to the conclusion that photosynthetic pigments have been enhanced by metallic nanoparticles. Because SeNPs preserve the enzymes in chloroplasts that are involved in the manufacture of chlorophyll, it is possible that this increase in photosynthetic pigments, and particularly in chlorophyll, is due to these nanoparticles, as selenium is considered to have a protective role for membranes and enzymes, explicitly against oxidative stress (Ragavan et al., 2017). According to the study conducted by Liang et al (Liang et al., 2020), selenium has the ability to improve chlorophyll florescence parameters, enhancing photosynthetic pigments along with maintenance of chloroplast ultrastructure. Enhancement in photosynthesis may be attributed to the maintenance of integrity of chloroplast membrane due to Se, hence causing less damage to chloroplast membranes (Kong et al., 2005). As low concentration of SeNPs has improved the chlorophyll content, higher concentration resulted in its decrease, which may be attributed to toxicity caused by SeNPs. Protein contains cysteine, and selenium binding to cysteine makes it Se–cysteine, so any increase in selenium concentration results in higher levels of Se–cysteine instead of cysteine only. Any new Se–Se diselenide bonding between protein or Se–S bonding results in disruption of the ultrastructure of photosystem II, resulting in reduced photosynthetic ability. Furthermore, it is also imperative to note that at lower concentration, selenium acts as a substituent for sulfur in the synthesis of chlorophyll, but as the concentration increases, the enzyme activity decreases, disrupting the chain of biochemical reaction and resulting in reduced chlorophyll content and net photosynthetic rate (Liang et al., 2020). It is also imperative to note that the decrease in photosynthetic pigments and photosynthesis is attributed to increased oxidative stress resulting in higher concentration of SeNPs, reducing chlorophyll syntheses, light absorption, low carbon assimilation, and ultimate reduction in growth and yield. Furthermore, it is also reported that selenium at higher concentration degrades chlorophyll precursor, resulting in reduced chlorophyll content; hence, it is very crucial to determine selenium concentration, as it may have negative effects on crop growth.

Reactive oxygen species (ROS) are essential for signaling in plants, but elevated levels result in oxidative damage to essential cellular organelles, resulting in disturbed functions. Accumulation of ROS mainly affects the membrane causing lipid peroxidation, membrane damage, impaired functions of important biomolecules like DNA and proteins, inhibition of the signal transductions pathways and even cause cell death. Enzymatic and non-enzymatic antioxidant defense systems found in plants work to scavenge ROS and keep their levels at optimum, protecting cellular machinery from oxidative damage. According to the current study, SeNPs have improved the antioxidant defense system, since SeNP concentrations were associated with higher activity of different enzymatic POD, SOD, APZ, GPX, and CAT (Shabala, 2017). Selenium is attributed the role of quenching ROS either directly or indirectly. A 55% increase in SOD activity was noted in SeNP-treated plants as compared to control, suggesting an increased SOD activity mediated by selenium. As SOD is responsible for quenching O_2_^•−^ to H_2_O_2_, there are three main factors that may control SOD activity in plants when exposed of selenium. One major factor is the dose of selenium given to plants; for example, a study conducted by Shabala (2017) concluded that aluminum-stressed ryegrass showed reduced SOD levels at 2 µM concentration of selenite supplementation, while at a concentration of 10 µM, the SOD activity increased (Shabala, 2017). This increased activity of these antioxidant enzymes is attributed to the selenium-mediated upregulation of genes responsible for antioxidant defense system enzymes. For example, a study conducted by Jiang et al., 2017 found that the activity of ZmMPK5 and ZmMPK7 and ZmCPK11 genes were upregulated in maize roots with application of selenium. These stress-responsive genes are activated by the levels of H_2_O_2_ and ultimate upregulation of antioxidant defense system in maize plants (Zhang et al., 2010). A similar conclusion was given by Ding et al., 2013 that the transient expression of ZmCPK11 has been shown to elevate both the expression and activity levels of antioxidant enzymes (SOD and APX) in maize.

Furthermore, sesame seed antioxidant parameters, i.e., DPPH, ABTS, TEAC, RPA, and TPC, were found to be significantly (*p ≤* 0.05) improved in SeNP-treated plants when compared to the untreated group. We found a dose-dependent effect of almost all the antioxidant parameters, suggesting a higher antioxidant potential of nanoselenium-treated plants. This improved chemical antioxidant ability of sesame seed may be attributed to metabolic improvement caused by treatments of SeNPs. Selenium or its derivative organic form can directly act as antioxidants and scavenging ROS, as this antioxidant activity is attributed to selenocysteine and selenomethionene, which is due to the nucleophilic properties of ionized selenol, along with ease of oxidation activity conferred by the these selenium-containing amino acids (Rahmanto and Davies, 2012). Furthermore, selenium-containing proteins and peptides extracted from plants were shown to have strong antioxidant activity, mainly the capacity to neutralize O2^•−^, DPPH^•^, and ABTS^+•^ radicals (Zhu et al., 2019; Zhang et al., 2020). In contrast to the Met-Pro-Ser peptide, a selenopeptide containing the SeMet-Pro-Ser sequence exhibited increased antioxidant activity (Liu et al., 2019). Research contrasting SeMet and Met’s antioxidant activity revealed that these amino acids have a similar mode of action when they come into contact with hydroxyl radicals. Nonetheless, Se-Met can oxidize more readily than Met due to its lower redox potential (Mishra et al., 2009). Thus, it can be inferred that the application of nanoselenium results in a higher content of selenium-containing amino acids and peptides, which directly confers the antioxidant activity in plants. Furthermore, anthocyanin, ascorbates, carotenoids, phenolics, and flavonoids are examples of primary and secondary metabolites that selenium can augment in plants, allowing for increased photosynthesis and sugar content (Golubkina et al., 2018). As phenolics and flavonoids have the ability to absorb free radical ions, thus selenium indirectly improves the non-enzymatic antioxidant defense system of plants by improving the metabolic profile (Jaiswal et al., 2018). Chemical antioxidants like phenolics, which were significantly improved with SeNP treatment (T4), have antioxidant activities due to which they reduce ROS, protecting essential biomolecules from oxidation and mitigating oxidative stress. This enhanced TPC activity may be attributed to the SeNP-mediated high nutrient availability, hence increasing the raw material along with improved activity of enzymes responsible for phenolic acids synthesis (Skrypnik et al., 2019). Another evidence in support was provided by, as they concluded that SeNPs improve Jasmonic acid content via alpha-linolenic acid pathway, inducing the synthesis and accumulation of phenolic and flavonoids in *Apium graveolens.* Authors also quantified the content of important phenolic and flavonoids in SeNP-treated and untreated plants and found that the content of coumaric acids, ferulic acids, apigenin, and rutin were increased by 80.4%, 68.2%, 58.4%, and 66.2% when compared to the control group (Li et al., 2020). Our results are consistent with the previous studies conducted by Bachiega et al., Puccinelli et al., and Ramos et al (Ramos et al., 2010; Bachiega et al., 2016; Puccinelli et al., 2017). The role of SeNPs in enhancing secondary metabolism was further confirmed by the study conducted by Rajaee Behbahani et al., 2020 that selenium nanoparticles upregulated the activity of *4CL* and *PAL* genes, hence with enhanced concentration of phenylalanine ammonia lyase enzyme, a critical enzyme responsible for synthesis of variety of secondary metabolites. Since the bulk of phenolic compounds found in plants are produced from phenylalanine, phenylalanine ammonia lyase (PAL) plays a crucial role in removing the ammonia and producing cinnamic acid. Therefore, increases in PAL content have a beneficial effect on the content of phenolic acids. It can be inferred that for the rise in phenolic acid content, is indirectly stimulated by selenium nanoparticles by elevating the levels of PAL, and ultimate enhancement of total secondary metabolites, especially soluble phenols.

Sesame being the “Queen of Oilseeds” is important for its high-quality oil, as the end product is the sesame seed or oil, which is utilized for various purposes. It was found that oil content in sesame was significantly improved by the application of SeNPs, as the highest percent increase was found in V1 at T5 SeNPs found to be 16.47% high as compared to untreated plants. This percent increase ranged from 9% to 16% in different treatments of SeNPs, when compared to untreated groups depending upon treatments and variety. Triacylglycerol synthesis shows involvement of two organelles, plastics and endoplasmic reticulum. The first part of TAG biosynthesis, the synthesis of fatty acids, is completed in plastics via the action of two critical and rate-limiting enzymes Acetyl coA carboxylase (ACC) and fatty acids synthase (FAS), while the second part, the assembly of these fatty acids occurs in the endoplasmic reticulum where the fatty acids are transported in the form of acyl-coA (Mishra et al., 2009; Kong et al., 2020). Various studies indicated the positive role of selenium in fatty acid desaturation and TAG accumulation, according to Kieliszek et al., 2019. *Saccharomyces cerevisiae* yeast supplemented with selenium showed an increase in the unsaturated fatty acid content (e.g., C18:1).

A crucial rate-limiting step in the fatty acid biosynthesis pathway is reached by the carboxylation of acetyl-CoA into malonyl-CoA, which is catalyzed by the key enzyme ACC (Sasaki and Nagano, 2004). According to the study conducted by Zhang et al (Zhang et al., 2024), the activity of ACC enzyme was increased by 37.1% by the application of selenium nanomaterials in *Glycine max.* In addition, the foliar application of Se nanomaterials markedly increased the relative expression of genes (FAD2-1A, FAD2-1B, and FAD3B) implicated in the oil synthesis pathway by 13.1%, 31.2%, and 7.2%, respectively. The study also concluded that selenium content was increased by 34.8% with decrease in sulfur content by 8.3%. As ACC is a sulfur-containing enzyme (Sasaki and Nagano, 2004), this enhanced activity may be attributed to the replacement of sulfur by selenium, as various studies proved that replacing S with Se improves the kinase activity (Sanmartin et al., 2011).

**Figure 9:**
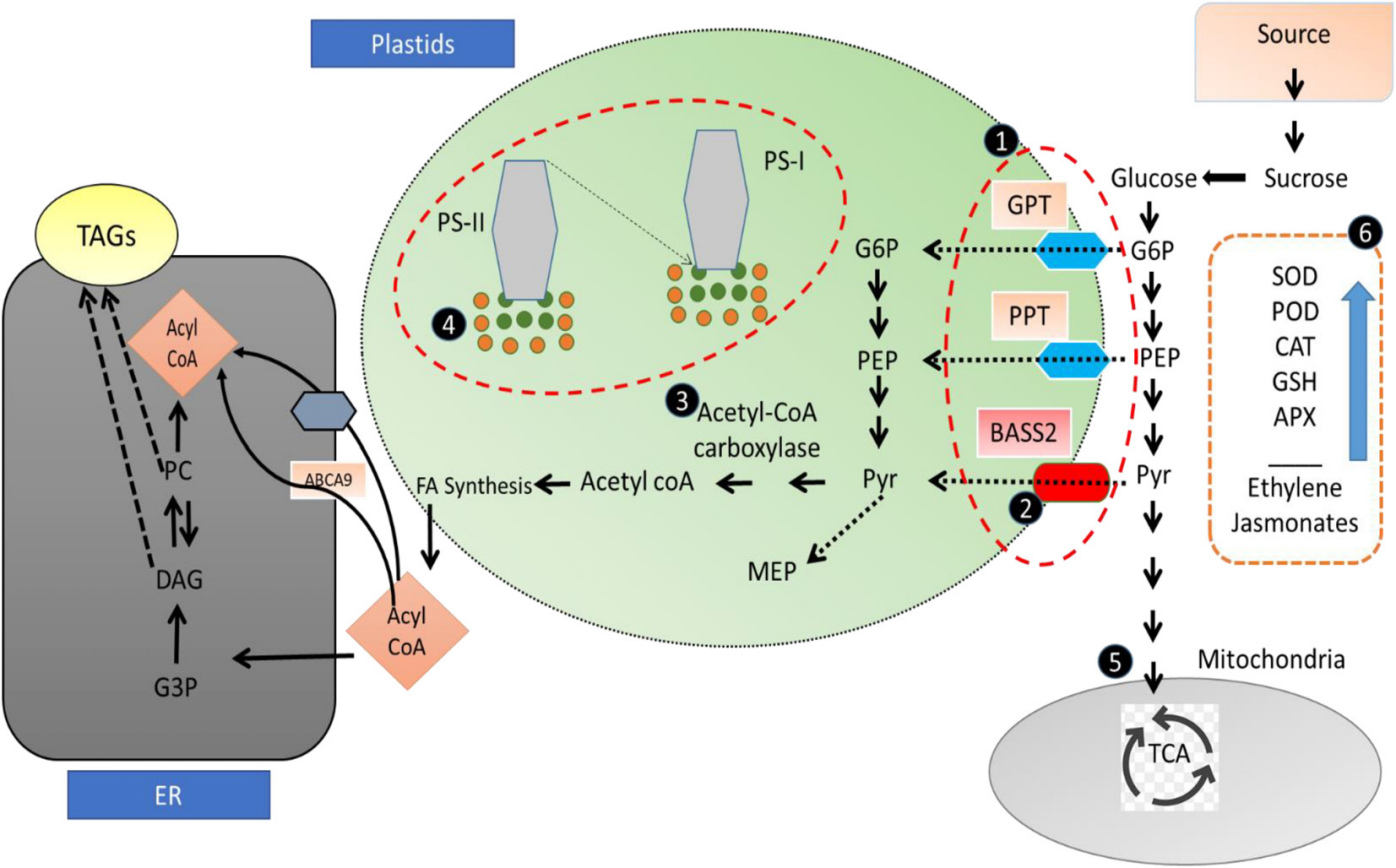
Proposed mechanism of selenium nanoparticles mediated growth and fatty acid improvement in sesame: (1, 2) glycerol 6 phosphate and phosphoenolpyruvate are transported to plastids by GPT and PPT transporters, embedded in the membrane, while selenium showed lowered MDA content in our results, hence with higher membrane stability and improved functioning of transporters, resulting in high content of raw material for oil biosynthesis. (3) Acetyl CoA carboxylase (catalyze rate-limiting step in oil biosynthesis) sulfur-dependent enzyme, its activity is enhanced when S is replaced with Se; in case of higher selenium content, sulfur is replaced with selenium, which exerts higher kinase activity of ACC enzyme, hence enhancing oil biosynthesis. (4) Selenium nanoparticles result in an improved photosynthetic pigment content (also shown I pour results) along with antioxidant role, thus preventing the oxidative damage to the photosynthetic machinery. Sugar and food provide raw material for oil biosynthesis; hence, SeNPs improve photosynthetic activity with higher photosynthetic product, which eventually feed for oil biosynthetic pathway. (5) Glycerol-3-phosphate (G-3-P) and acetyl-CoA produced by glycolysis/gluconeogenesis are the raw materials for FA biosynthesis and TAG assembly, indicating that carbohydrate metabolism may have important implications for the improvement of oil content in plants. (6) As SeNPs have also been reported to have an upregulation of enzymatic antioxidant defense system and hormone concentration resulting in overall growth and oil yield improvement.

Therefore, on the basis of these results, it can also be concluded that the sesame-enhanced oil accumulation in nanoselenium-treated plants is due to the enhanced activity of the rate-limiting enzyme ACC and upregulating the expression of DGAT genes involved in the sesame oil synthesis pathway, and the expression of FAD2-1A, FAD2-1B, and FAD3B genes associated with the fatty acid desaturation process (**Figure 9**).

Furthermore, the indirect role of selenium nanoparticle for oil enhancement may be attributed to its different biological functions, like enhancement of antioxidant defense system, protection of cellular membranes, protection of photosynthetic machinery, and enhanced food production, hence providing sufficient rat material for synthesis of oil in sesame. As most of the carriers and transporters are embedded in biological membranes, SeNPs, by reducing lipid peroxidation, give stability and hence higher activity of these transporters for transporting raw material for oil fatty acid biosynthesis. Furthermore, sugars are the major raw material for synthesis of fatty acids, as selenium improves pigment content and gives protection to photosynthetic machinery, hence with higher food production and more food for oil biosynthesis.

## 6 Conclusion

In conclusion, the findings of this study underscore the significant potential of plant-mediated selenium nanoparticles (SeNPs) in enhancing the growth, physiological processes, and oil yield of sesame (*Sesamum indicum* L.). Through a comprehensive investigation involving various SeNP treatments and application methods across six sesame varieties, it was evident that the seed + foliar treatment method, particularly at concentrations of 40 and 50 ppm (T4 and T5), exhibited the most pronounced positive effects. The application of SeNPs resulted in notable improvements in agronomic parameters, chlorophyll content, antioxidant activity, and triacylglycerol (TAG) profile, with significant variations observed among the sesame varieties. Furthermore, UHPLC-MS analysis and chemometric modeling identified 10 TAG biomarkers, indicating a potential mechanism underlying the enhanced oil yield in SeNP-treated sesame. Overall, the study highlights the promising role of SeNPs in sesame cultivation, particularly when applied through the seed + foliar treatment method at optimized concentrations. It is also imperative to note that although SeNPs improved growth attributes, the effect was not homogeneous in all the varieties, suggesting that there is an interaction between the germplasm and SeNPs. It also suggests that the effectiveness of nanoparticles depends upon the dose and germplasm, which, in combination, results in the effectiveness of the treatments. These findings not only contribute to our understanding of the potential benefits of SeNPs in agriculture but also emphasize the need for further transcriptomic and molecular analyses to elucidate the underlying mechanisms driving the observed improvements. Further research in this area is warranted to fully harness the potential of SeNPs in enhancing crop productivity and oil yield in sesame and other agricultural crops.

## Data Availability

The original contributions presented in the study are included in the article/supplementary material. Further inquiries can be directed to the corresponding authors.
